# Satellite data lift the veil on offshore platforms in the South China Sea

**DOI:** 10.1038/srep33623

**Published:** 2016-09-19

**Authors:** Yongxue Liu, Chao Sun, Jiaqi Sun, Hongyi Li, Wenfeng Zhan, Yuhao Yang, Siyu Zhang

**Affiliations:** 1 Department of Geographic Information Science, Nanjing University, Nanjing, 210023, P. R. China; 2 Collaborative Innovation Center for the South China Sea Studies, Nanjing University, Nanjing, Jiangsu Province 210023, P. R. China; 3 International Institute for Earth System Science, Nanjing University, Nanjing, 210023, P. R. China; 4 Jiangsu Center for Collaborative Innovation in Geographical Information Resource Development and Application, Nanjing, 210023, P. R. China

## Abstract

Oil and gas exploration in the South China Sea (SCS) has garnered global attention recently; however, uncertainty regarding the accurate number of offshore platforms in the SCS, let alone their detailed spatial distribution and dynamic change, may lead to significant misjudgment of the true status of offshore hydrocarbon production in the region. Using both fresh and archived space-borne images with multiple resolutions, we enumerated the number, distribution, and annual rate of increase of offshore platforms across the SCS. Our results show that: (*1*) a total of 1082 platforms are present in the SCS, mainly located in shallow-water; and (*2*) offshore oil/gas exploitation in the SCS is increasing in intensity and advancing from shallow to deep water, and even to ultra-deep-water. Nevertheless, our findings suggest that oil and gas exploration in the SCS may have been over-estimated by one-third in previous reports. However, this overestimation does not imply any amelioration of the potential for future maritime disputes, since the rate of increase of platforms in disputed waters is twice that in undisputed waters.

Offshore oil/gas platforms are the essential infrastructure for the extraction, processing, and temporary storage of crude oil and natural gas in the sea. The question of exactly how many oil and gas offshore platforms are distributed in the South China Sea (SCS) is of significant interest both for academic research and economic and geopolitical considerations, and from national level to trans-national level: (*1*) Understanding the regional distribution of the offshore associated petroleum gas (APG) flaring is essential for the drive to assess APG utilization led by the Global Gas Flaring Reduction Partnership (GGFR)^1^. (*2*) Energy resources are an undeniable factor underlying sovereignty and maritime disputes in the SCS, and tension is growing in line with increasing offshore energy exploitation^2^,^3^. Accurate knowledge of the total number of platforms and its rate of change may aid the SCS claimants to better manage their safety and security risks. (*3*) More than one half of the world’s oil tankers and merchant ships pass through the SCS every year^4^, and detailed knowledge of the locations of the platforms will reduce the potential for collisions within this vital sea lane^5^. (*4*) Offshore platforms promote neighboring secondary production^6^,^7^, but they also pose potentially serious risks to the marine environment^8^,^9^,^10^,^11^,^12^, especially from oil spillages^13^,^14^,^15^,^16^. Moreover, the growing number of aging platforms, approaching or exceeding their design life, increases the potential for environmental hazards, and knowledge of the ages of platforms will facilitate screening of high-risk areas. (*5*) Many platforms are equipped with rescue facilities (*e.g.*, stand-by vessels and helipads), and knowledge of the types of platforms may be valuable when preparing for futures hazards such as tsunamis.

Admittedly, the relevant authorities, such as offshore oil/gas producing countries, offshore energy producers, and even offshore platform producers, are familiar with the details of their own platforms. However, they are usually reluctant to make this information available publically–for reasons of business secrecy or national interests and security. This creates transnational knowledge barriers, and even state powers may lack timely and complete information concerning offshore platforms located in neighboring waters. For example, the Ministry of Defense of Japan has had to resort to aerial reconnaissance in order to assess the spatial distribution of platforms in the East China Sea^17^. Similarly, related parties of the SCS are confronted by the same dilemma, and there are various opinions regarding the number of offshore oil/gas platforms in the SCS^18^,^19^,^20^,^21^. Thus, the uncertainty with regard to the total number of offshore platforms, let alone their detailed spatial distribution and dynamic changes, may lead to significant misjudgment of the true status of hydrocarbon production in the SCS. Earth observation satellites provide remote and regular access to inaccessible areas^22^,^23^, and the large amount of new and archived satellite data, most of which is free and web enabled^24^,^25^,^26^, and which is continuously improving in spatial, temporal and spectral resolution, provides the opportunity to overcome the knowledge barrier.

In order to enable non-professionals, including managers and decision-makers, to refine their strategies for marine energy exploitation, environmental management, and marine security/safety maintenance, we focus primarily on revealing the exact number, spatial distribution, rate of increase and type of oil and gas platforms in the SCS via analysis of time series satellite images with a variety of spatial resolutions.

## Results

### Offshore persistent-flaring regions detected from the nighttime light (NTL) products

A total of 112 offshore persistent-flaring regions (flaring consecutively for more than two years) in the SCS were determined from the annual NTL products generated from the operational line-scan system (OLS) of Defense Meteorological Satellite Program satellites (DMSP). These flaring regions are distributed on the continental shelf off the mouth of the Pearl River and the Gulf of Tonkin, the southeast coast of the Indo-China Peninsula, the region from the Gulf of Thailand to the west Natuna Islands, the north coast of Kalimantan Island, and the west coast of the Philippine Islands (Supplementary Fig. S1). Offshore persistent flaring regions derived from the monthly NTL products acquired by the visible infrared imaging radiometer suite (VIIRS) boarded on the Suomi National Polar-orbiting Partnership (S-NPP) weather satellite exhibit a remarkably similar spatial distribution (Supplementary Fig. S2). Although it is impossible to determine accurately the number and exact position of platforms from NTL products because of their coarse spatial resolution, the distribution of persistent flaring regions overall provides valuable heuristic information for more detailed platform detection in the entire SCS.

### Up-to-date distribution of offshore platforms detected from moderate resolution images

**Figure 1:**
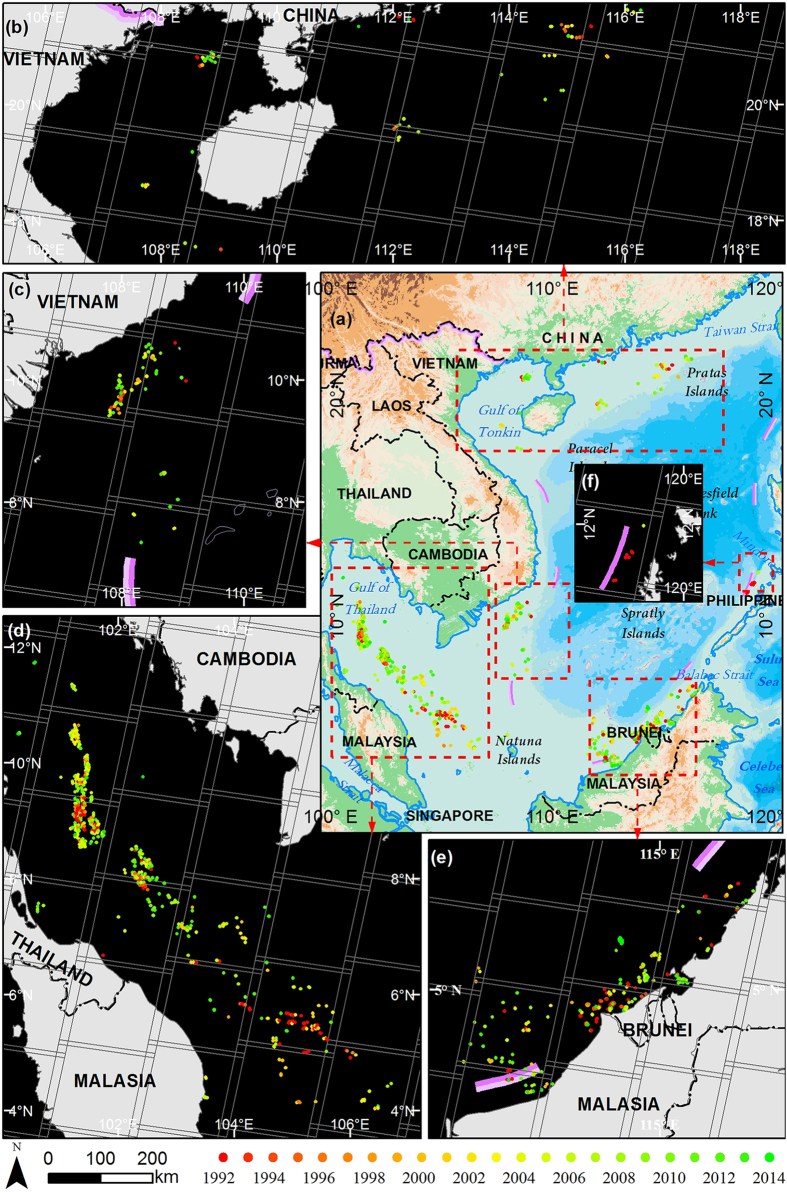
Annual proliferation of offshore platforms in the SCS from 1992 to 2015. (**a**) The entire SCS; (**b**) Gulf of Tonkin and offshore of the Pearl River mouth; (**c**) the southeast coast offshore of the Indo-China Peninsula; (**d**) from the Gulf of Thailand to the west of the Natuna Islands; (**e**) the north coast offshore of Kalimantan Island; (**f**) the coast offshore of the Philippine Islands. The map data was made with Natural Earth (http://www.naturalearthdata.com/). The figure was generated by Y. L. and W. C. using ArcMap 10.0 (http://www.esrichina.com.cn/).

Our detection shows there were a total of 1082 platforms across the SCS by March 2015 (Fig. 1). Among these platforms, 1040 platforms were detected from 1059 candidates retrieved from more than a thousand Landsat-8 operational land imager (OLI) images between 2013 and 2015 (with 19 were incorrectly detected), and the rest 42 platforms were detected based on other remote sensing data ranging from SAR to high-resolution optical images (Supplementary: data sets). The elaborate crosscheck illustrates that the omission error and the commission rate are 3.88% and 1.76%, respectively (Supplementary: validation). After the refinement by the use of multi-source data across the entire SCS, the accuracy of this number (1082) is likely to be robust, because the omissions and commissions confirmed by those time-series images from various sensors have been further corrected.

The spatial distribution of the detected platforms accords well with that of the persistent flaring regions (Supplementary Figs S1 and S2). Combining our results with the marine claims made by the littoral states of the SCS, we determined that Thailand had the greatest number of platforms (356), followed by Malaysia (317), Brunei (166), Vietnam (91), China (76), Indonesia (29), Philippine (8), Cambodia (1), the Malaysian-Thai Joint Development Area (MTJDA, 25), and the Malaysian-Vietnamese Joint Economic Development Zone (MVJEDZ) (13).

Satellite images and bathymetry data reveal the majority of oil and gas exploitation in the SCS is characterized by small-installation and shallow-water production (Supplementary Figs S3–S5). We found that a total of 892 platforms were single-structure and of small/medium size; 81 were hybrid structures comprising several sub-platforms; and 109 were ship-shaped, presumably floating production storage and offloading units (FPSO) (Supplementary Fig. S4). More than 95% of platforms (1031) were located in shallow water (<100 m water-depth), 40 installations were located in water depths from 100 to 500 m, and only 11 were deep-water platforms (>500 m water-depth) (Supplementary Fig. S5).

### Proliferation of offshore platforms documented in archived moderate resolution images

**Figure 2:**
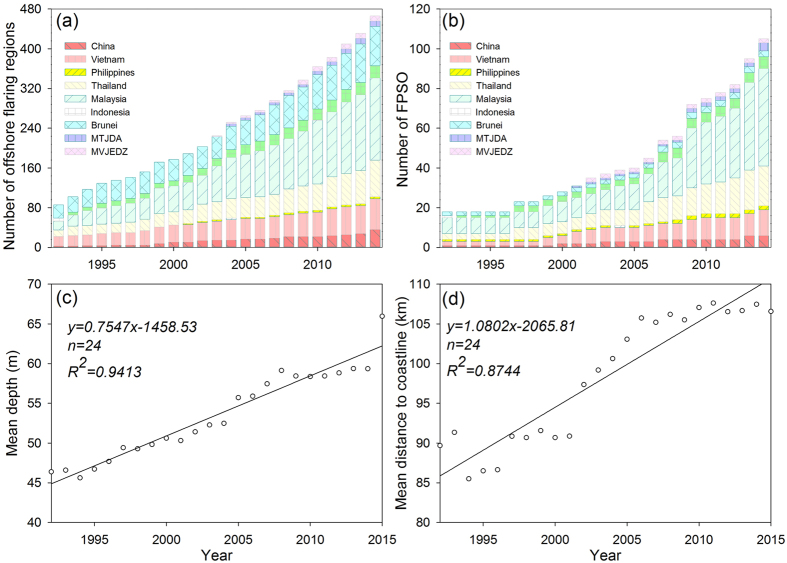
Annual proliferation of offshore platforms in the SCS. (**a**) Annual number of platforms activating at nighttime; (**b**) annual number of FPSO; (**c**) annual mean depth of offshore platforms; and (**d**) annual mean distance of offshore platforms to the mainland. The figure was generated by Y. L. J. S., and W. C. using SigmaPlot 12.5 (http://www.sigmaplot.com/).

According to more than 4,000 archived images acquired from 1991 to 2015 (see Supplementary: data sets for more detail), we detected a continuous increase in offshore hydrocarbon exploitation in the SCS: (*i*) The number of offshore platforms in the SCS increased steadily from 230 in 1992 to 1082 in 2015–an average of 37 new platforms per year, and a rate of increase approximately 4.7 greater than during the two previous decades (Supplementary Table S1 and Supplementary Fig. S6). (*ii*) By overlapping the distribution of annual persistent flaring regions detected from NTL products with our detection results, we determined that the number of offshore platforms operating at night maintained a higher growth rate, with the number increasing from 86 to 466, an increase by a factor of 5.41 (Fig. 2a). (*iii*) There was a similar trend in the number of FPSO, ranging from 18 in 1992 to 109 in 2015, an increase by a factor of 6.06 (Fig. 2b). The data demonstrate that the pattern of offshore oil/gas exploration and exploitation in the SCS exhibits consistency in terms of operational depth as well as in the distance to the mainland (Fig. 2c,d). (*iv*) The operational depth ranges from shallow water to deep water, and even approaches ultra-deep water (>1500 m), with the maximum depth increasing from approximately 119 m in 1992 to 1469 m in 2015. (*v*) The distance to the mainland ranged continuously from near-shore to open sea, with several platforms located more than 340 km from the mainland.

## Discussion

### Possible overestimation of the number of offshore platforms in previous reports

Until now, there was no available spatial distribution data of offshore platforms across the SCS, and the precise number of offshore platforms over the region remained in dispute. Several reports claimed that approximately 1278^18^, 1350^19^, 1380^20^ platforms had been erected in the SCS by 2010, while later reports asserted that the number had increased to 1511 by August 2015^21^. All of these previous reported numbers were primarily obtained by integrations, estimations, and speculations from decommissioned studies, proprietary and commercial databases, and Internet searches. They vary as a result of data availability, classification criteria of platforms, and date of assessment^19^.

**Figure 3:**
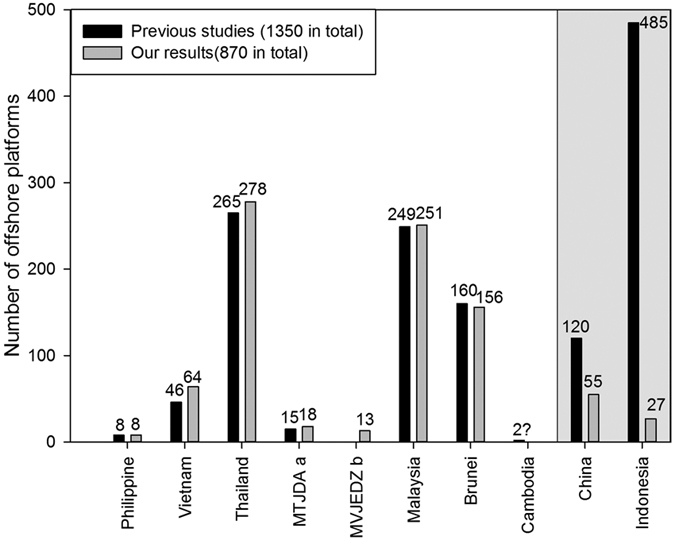
Comparison of the number of offshore platforms distributed in the SCS by 2010 between our results and that reported in the previous studies. The figure was generated by Y. L., J. S. and W. C. using SigmaPlot 12.5 (http://www.sigmaplot.com/).

In contrast, we enumerated a total of 870 platforms by 2010 from archived time-series images covering the entire SCS (Supplementary Table S1). This number is significantly lower than the numbers in previous reports. However, in a country-by-country comparison, the number of platforms belonging to most of the SCS claimants/joint-development-areas, including Philippine, Vietnam, Thailand, Malaysia, MTJDA, and MVJEDZ, are generally consistent or slightly higher than those previously reported (Fig. 3). We consider that a timely update of these earlier reports across the SCS is lacking, because every offshore platform we detected has been convincingly evidenced by time-series satellite images (Supplementary: validation).

**Figure 4:**
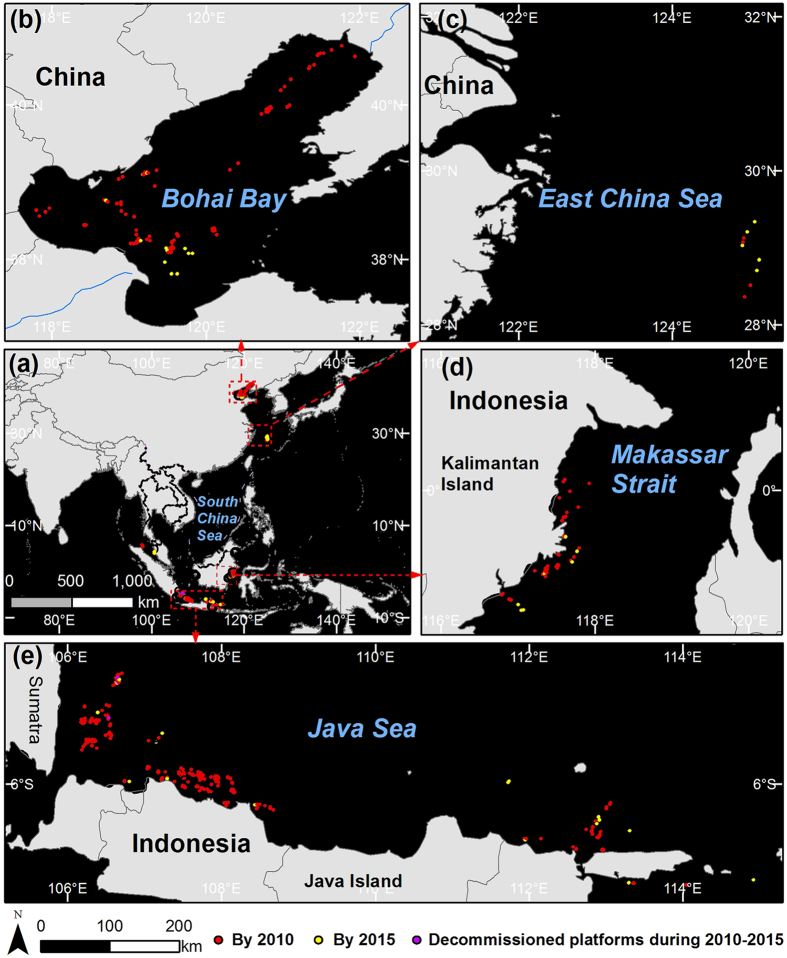
Offshore platforms outside the SCS (**a**). Platforms owned by China in Bohai Bay (**b**) and in the East China Sea (**c**); platforms owned by Indonesia offshore the east Kalimantan Island (**d**) and offshore the Sumatra Island and Java Island (**e**). The map data was made with Natural Earth (http://www.naturalearthdata.com/). The figure was generated by Y. L. using ArcMap 10.0 (http://www.esrichina.com.cn/).

The most striking divergence between previous results and our own findings occurs in the case of China and Indonesia: the number of offshore platforms we detected belonging to China is less than half that previously reported, and the number belonging to Indonesia is approximately one-twentieth of that previously reported. We suggest that the data in the previous reports may include platforms outside the SCS but which are owned by the SCS claimants. Evidence in support of this conclusion is the fact that three types of quasi-synchronous space-borne image, acquired by Landsat-5/7 TM/ETM+, ALOS-1 PALSAR, and ENVISAT ASAR, covering all the areas of Chinese exploitation in the SCS, indicate that the number was no more than 55. According to our extended remote sensing monitoring, by 2010 the total of offshore platforms in the Bohai Bay (87, Fig. 4b), in the East China Sea (4, Fig. 4c), and the SCS (55, Fig. 1b), was 146. Further evidence is provided by the fact that by 2010 the total will reach 1256, excluding the offshore platforms owned by Indonesia but which lie outside the SCS (Fig. 4d,e), such as the water to the east of Kalimantan Island (42, Fig. 4d), south of Sumatra and Java (253, Fig. 4e). These regions are not geographically part of the SCS (Fig. 4a).

Even taking into account the fact of statistical region, the following two factors may also result in an overestimation: (*i*) *Decommissioned platforms* – Our results show that at least 290 platforms across the SCS have been operating for more than 20 years (Fig. 1 and Supplementary Table S1), approaching their designed service life (25-year). These decommissioned platforms may not have been excluded in previous reports. For example, in the sea area claimed by Brunei, 23 scenes of GF-1/ZY-3 high resolution images covering the entire region indicate that the number was no more than 156 (Supplementary: validation), slightly less than the number of 160 reported in a previous study. In addition, we determined that at least four platforms had been removed from 2007–2009, which may explain the divergence. *(ii) Classification criteria* – Our results demonstrate that there were 81 hybrid platforms consisting of several sub-platforms in the SCS (Supplementary Fig. S4). These complex structures have been expanding for several years (Supplementary Fig. S7), resulting in their being enumerated several times.

We consider that the combination of these factors, i.e., region of the statistics, decommissioned platforms, and classification criteria, has likely resulted in a roughly one-third overestimation of the number of platforms in the SCS.

### Future geopolitical tension caused by oil and gas exploitation in the SCS

Clearly, overestimation of the number of offshore platforms in the SCS does not imply any future amelioration in maritime tension caused by the proliferation of offshore oil and gas exploitation in the region. Our data reveal that by March 2015, 90 platforms were located in regions of overlapping maritime claims, almost 10 times the number in 1992 (Supplementary Fig. S8). This rate of increase in disputed waters was more than twice that in undisputed waters. We predict that by 2020 the number of offshore platforms in areas of overlapping claims will reach 120, based on the average of 6 new platforms per year during the past decade, and approximately 130 based on the average of 8 during the past five years.

Obviously, the proliferation of offshore oil and gas exploitation will continuously intensify maritime tensions in the SCS, and this situation is likely to be further exacerbated with the surging domestic demand and the depletion of inshore hydrocarbon energy resources of the littoral states of the SCS^2^. Furthermore, the SCS is an area of major and long-term economic and strategic interest for several major powers, and the overlapping factors of sovereignty disputes and energy aspirations, as well as the intertwining of the political and economic interests of neighboring countries and the strategic involvement of external countries, result in considerable geopolitical complexity^27^,^28^,^29^. Therefore, disputes triggered by the proliferation of offshore oil and gas exploitation may escalate to conflicts which are grounded in efforts to maintain national sovereignty which transcend the desire simply to maintain oil and gas production. Such conflicts may potentially lead to serious international crises.

## Data and Methods

**Figure 5:**
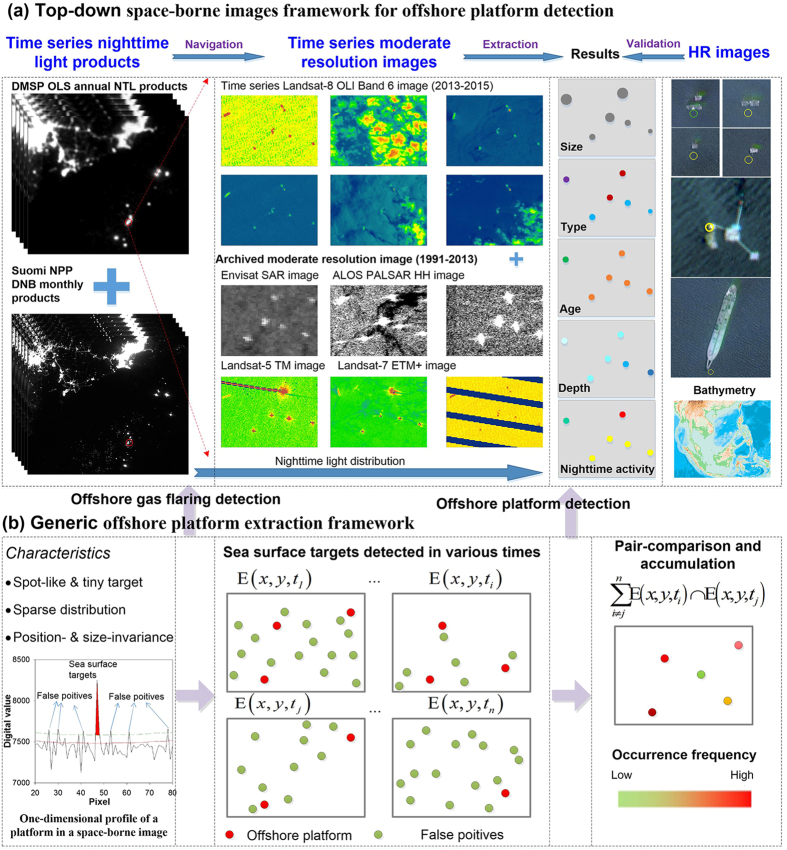
The proposed data framework and general framework for offshore platform detection. The DMSP OLS NTL data was downloaded from the NGDC Earth Observation Group (EOG) of NOAA (http://ngdc.noaa.gov/eog/download.html). The Landsat-8 OLI SWIR data were available from the Global Visualization Viewer of the U.S. Geological Survey (http://glovis.usgs.gov). The ALOS PALSAR Standard Product ((**c**) JAXA/METI) were downloaded from the Japan Aerospace Exploration Agency (JAXA, http://www.eorc.jaxa.jp/). The GF-1 images were downloaded from the China Center for Resource Satellite Data and Applications (CRESDA, China, http://www.cresda.com/). The figure was generated by Y. L. using ArcMap 10.0 (http://www.esrichina.com.cn/) and Office Visio 2010 (https://products.office.com/en-us/visio).

The enumeration of offshore platforms in the SCS, an area of more than 3.8 million km^2^ (Supplementary: the South China Sea), by means of remote sensing (often influenced by poor image conditions, complex underlying surface, and ubiquitous noise), is problematic. To address the aforementioned difficulties, a top-down data framework incorporating macro/meso/micro scales of satellite images, and a generic offshore platforms detection method for various capable space-borne sensors, was designed (Fig. 5).

### Top-down space-borne data framework for offshore platform detection

The past unsuccessful search for Flight MH370 highlights the fact that the search for marine targets, e.g., plane debris, should be based on the premise of coarse/fine location information^30^. Similarly, pre-knowledge of offshore platforms, e.g., their approximate but timely spatial-distribution, is critical for their detection. Hence, a multi-resolution space-borne image framework was designed to efficiently detect offshore platforms across a large area of sea (Fig. 5a):

1. *Heuristic information derived from low resolution nighttime light products –* Gas flaring of APG and lights from offshore platforms at night can be recorded in the DMSP OLS, or in the VIIRS boarded on the S-NPP weather satellite^31^,^32^. Thus, the persistent flaring regions in time-series NTL images, reflecting the timely and coarse distribution of platforms, can be used as an indicator for determining which moderate-scale sensors are qualified for offshore platform detection, and for which areas moderate resolution images need to be collected.
2. *Detailed information derived from competent moderate resolution images –* Using NTL products for guidance, we find that Synthetic Aperture Radar (SAR) images acquired by ENVISAT, ALOS, Sentinel, and the like, and short wave infrared (SWIR) images photographed by Landsat thematic mapper (TM), enhanced thematic mapper plus (ETM+), and operational land imager (OLI), are capable of presenting offshore platforms. Note that the aforementioned sensors vary greatly in terms of available time-span, spatial coverage, archived number, geometric accuracy, and cost of space-borne imagery (Supplementary Table S2). After considering their merits and demerits, we selected Landsat-8 OLI images, which offer the latest and almost complete coverage of the SCS, to detect the up-to-date distribution of platforms in the SCS, while employing the other archived images to determine the ages of offshore platforms (the year of first appearance). Furthermore, by combining platform positions with bathymetry data, the water depth of offshore platforms can also be retrieved.
3. *Validation from high resolution images –* High resolution images from the Chinese satellites GF-1 and ZY-3, were used to validate and refine the detections from moderate resolution images.

According to the aforementioned multi-resolution space-borne image framework, a total of more than 4,000 satellite images covering the SCS were used, including 1035 Landsat-8 OLI images (2013–2015), 1196 Landsat-5/7 TM/ETM + images (1991–2013), 1280 tiles ALOS-1 PALSAR images (2007–2010), 573 ENVISAT ASAR images (2005–2009), 86 Sentinel SAR images (2014–2015), and 89 images from Chinese high resolution satellites GF-1 and ZY-3. More information on the data used, such as the available time-span, spatial coverage and number, is given in Supplementary: data sets.

### Generic platform extraction method for various capable sensors

Offshore platforms documented in the competent space-borne images, usually exhibit the following characteristics: *(1) Tiny, spot-like features with a sparse distribution* (Fig. 5a). (2) *Invariance characteristics–*In a time-series of satellite images with high geometric accuracy, the positions of offshore gas-flaring/platforms can be regarded as almost fixed relative to moving vessels. According to these two characteristics, we extend the automated method for extracting offshore platforms (AMEOP) from time-series OLI images which we proposed previously^33^, to time-series satellite images acquired by other capable sensors (Fig. 5b):

1. *Detecting offshore platform candidates according to spot-like principles –* Sea surface targets, including platforms, vessels, and noise, usually exhibiting a homogeneous compact region in competent images, can be regarded as salient points with DN values higher than their surroundings. These salient targets, striking or not, can be segmented using an adaptive threshold calculated by order-statistic filtering (OSF). Admittedly, not all offshore platform candidates can be completely extracted from a single phase satellite image because of weak imaging conditions, or the influence of noise. We detect offshore platform candidates from a time-series of images acquired in a short time span to minimize the omissions: the undetected platform candidates in a given phase would be compensated in other phase images with high quality.
2. *Excluding false positives according to the position invariance principle –* Usually, noise among the candidates was randomly distributed in the spatiotemporal dimension, and we apply pairwise intersection to the time-series of offshore platform candidates to mitigate the mixed errors. In each pairwise intersection, some offshore platforms may be improperly removed because they are undetected in paired images, or some noise may be retained because of the over-density of noise in coupled images. Subsequently, all pairwise intersections were accumulated to compensate for omissions and to suppress noise. In the accumulation image, the offshore platforms (with a high degree of position invariance in image time-series) usually had an occurrence frequency higher than that of randomly-distributed noise sources. Thus, those pixels with high frequency were segmented as potential offshore platforms.
3. *Confirmation –* To maximize the robustness of the detection, all platforms automatically detected from time-series images were then repeatedly examined and carefully assessed by time-series of high quality Landsat-8 OLI images, ALOS PALSAR validation all over the entire SCS, Sentinel-1 SAR validation along the coastal zones, and high resolution image validation in the coastal zones and areas of offshore platform agglomeration. In addition, in the process of manual validation, the type of offshore platform was determined as a byproduct according to their shape exhibited in the Landsat-8 OLI images.

Clearly, the position-invariance characteristic of offshore platforms/gas flaring regions documented in the time-series images is of vital importance for their detection, and the first two steps mentioned above can be automatically applied to competent satellite images with eligible geometric accuracy (e.g., <2 pixels), such as images acquired by DMSP OLS, S-NPP VIIRS, Landsat-8 OLI, and ALOS-1 PALSAR. Other competent images with a poor geometric accuracy, e.g., Landsat TM/ETM+, ENVISAT ASAR images covering areas far from land in which the shift of the same platform may span dozens of pixels, were geometric-corrected to ensure their position-invariance, using the platforms detected from Landsat-8 images as ground control points. More details on the generic platform extraction method for various capable sensors are given in Supplementary: Table S3, Figs S11–S16, and Method for offshore platform detection.

## Additional Information

**How to cite this article**: Liu, Y. *et al.* Satellite data lift the veil on offshore platforms in the South China Sea. *Sci. Rep.* **6**, 33623; doi: 10.1038/srep33623 (2016).

## Supplementary Material

Supplementary Information
