# Supplementary material for: Satellite data lift the veil on offshore platforms in the South China Sea
Source: Sci Rep. 2016 Sep 19;6:33623. doi: 10.1038/srep33623 (PMC5027537; doi:10.1038/srep33623)
Supplement: Supplementary Information [file srep33623-s1.pdf]

Satellite data lift the veil on offshore platforms in the South China Sea

Yongxue Liu[1,2,4*], Chao Sun[1], Jiaqi Sun[1], Hongyi Li[1], Wenfeng Zhan[3], Yuhao Yang[1], and Siyu Zhang[1]

Supplementary Table 1 | **Summary of the number of offshore platforms in the SCS between 1992 and 2015**

Year	SCS	China	Vietnam	Philippine	Thailand	Malaysia	Indonesia	Brunei	Cambodia	MTJDA [a]	MVJEDZ [b]
1992	230	9	15	7	52	82	4	61	0	0	0
1993	243	11	19	7	57	84	4	61	0	0	0
1994	272	12	19	7	62	94	4	74	0	0	0
1995	290	15	21	7	69	98	4	76	0	0	0
1996	304	19	22	7	70	103	4	79	0	0	0
1997	349	19	23	7	97	108	10	85	0	0	0
1998	350	19	23	7	97	109	10	85	0	0	0
1999	378	21	30	7	98	118	10	93	0	1	0
2000	402	26	30	7	98	130	10	100	0	1	0
2001	440	26	37	7	99	144	11	109	0	7	0
2002	484	28	42	7	114	157	13	111	0	7	5
2003	523	34	44	7	125	165	20	115	0	7	6
2004	549	36	45	7	137	172	20	118	0	7	7
2005	584	39	46	7	150	183	20	123	0	7	9
2006	641	42	50	7	191	187	20	127	0	7	10
2007	727	48	52	7	215	215	24	145	0	10	11
2008	763	52	54	8	231	221	24	149	0	13	11
2009	829	55	61	8	252	244	26	156	0	14	13
2010	870	55	64	8	278	251	27	156	0	18	13
2011	898	58	67	8	293	259	27	155	0	18	13
2012	929	61	72	8	304	264	27	162	0	18	13
2013	1014	66	80	8	339	295	28	162	0	23	13
2014	1060	74	90	8	350	307	29	164	0	25	13
2015	1082	76	91	8	356	317	29	166	1	25	13

a. MTJDA – Malaysian-Thai Joint Development Area.

b. MVJEDZ – Malaysian-Vietnamese Joint Economic Development Zone.

Supplementary Table 2 | **Comparison of satellite images/products used in this study**

**Satellite Sensor**	**Avaiable Period**	**Coverage**	**Resolution**	**Geometric Accuracy**[a]	**Archieved images**	**Cost**	**Purpose**
DMSP OLS	1992-2013	All covering	30 arcseconds	≤ 3 pixel	34 [b]	Free	To get coarse location of offshore platforms
S-NPP VIIRS	2012-2015	All covering	15 arcseconds	≤ 2 pixel	16 [c]	Free	
Landsat-8 OLI	2013-2015	≈All covering	30 meters	≤ 2 pixels	≥ 15 tiles	Free	To get up-date, exact distribution in 2015
Landsat-4/5 TM	1984–2013	≈All covering	30 meters	≤ 5 pixels	≥ 5 per tile per year	Free	
Landsat-7 ETM+	1999–2015[d]	≈All covering	30 meters	≤ 3 pixels		Free	To get distribution during 1992-2014
Envisat ASAR	2005–2008	Coastal zone	150 meters	≤ 5 pixels		Free	
ALOS PALSAR	2006-2011	All covering	25 meters	≤ 3 pixels		Free	
Sentinel-1 C-SAR	2014-2015	Coastal zone [e]	20 meters	≤ 2 pixels	2-3 per tile	Free	
GF-1 CCD	2013-2015	Local covering	2 meters	≤ 8 pixels			For validation
ZY-3 CCD	2012-2015	Local covering	6 meters	≤ 6 pixels			

a. There are rarely quantitative assessments of the geometric accuracy of satellite images over sea, and the number listed in the table is the majority offset distance of the same offshore platform/ offshore flaring existing in pairwise images according to our observations. In some cases, the offset, e.g., the Landsat-5 TM image photographed on November 2, 2011 (path/row: 128/053), can span more than 20 pixels (i.e., 600 m).

b. A total of 34 yearly nighttime light products have been generated from daily images photographed by six DMSP satellites (F10, F12, F14, F15, F16, and F18) spanning the period of 1992-2013 by NGDC Earth Observation Group (EOG) of NOAA (http://ngdc.noaa.gov/eog/, accessed 19th July 2015).

c. A total of 16 monthly S-NPP VIIRS DNB nighttime products have been generated by EOG (http://ngdc.noaa.gov/eog/, accessed 19th July 2015).

d. The Scan Line Corrector (SLC) in the ETM+ instrument failed on May 31, 2003.

e. The Sentinel-1, launched on 03 April 2014, provides 250 km Swath of C-band Synthetic Aperture Radar (C-SAR) in Interferometric Wide Swath Mode covering. To date, almost all available Sentinel-1 data only cover the coastal zone of the SCS.

Supplementary Table 3 | **Parameters used in the automated method to identify offshore platforms for the space-borne images**.

Step	Parameters name	OLS	DNB	OLI	PALSAR
Sea surface targets detection<br>$E\left(x,y,t_i\right)=\begin{cases}1, IF\ f_s(x,y,t_i)>0\\0, Otherwise\end{cases}$<br>Where<br>$f_s(x,y,t)= f(x,y,t)- f_b(x,y,t)- f_n(x,y,t)$<br>$f_b(x,y,t_i)\approx \underset{u,v}{r^{th}\text{-}OSF}\left(f\left(x-u,y-v,t_i\right)+w\left(u,v,t_i\right)\right)$<br>$f_n(x,y,t)\approx \underset{u,v}{STD}(f(x\text{-}u,y\text{-}v,t)+w(u,v,t))+\xi$	*n*	2	16	11	2
	MS	Disk	Disk	Disk	Disk
	MR	20	20	12	20
	r^{th}	50% (median)	50% (median)	90%	50% (median)
	ζ	6	10	20	800
	Area	-	-	<550	-
		-	-	The detected sea surface targets were dilated by mathematical morphology transformation with a disk-structure element (radius = 1 pixels) to mitigate the influence by image geometric accuracy	
Noise removal<br>$E_{ij}=E\left(x,y,t_i\right)\cap E\left(x,y,t_j\right)$				-	-
		-	-	Intersections of pairwise sea surface targets were dilated by mathematical morphology transformation with a disk-structure element (radius = 1 pixels) to mitigate the influence by image geometric accuracy	
Platform segmentation<br>$P\left(x,y,t_i\right)=\begin{cases}1, IF\ \text{Frequency}(x,y)>T\\0, Otherwise\end{cases}$	$m=C_n^2$	1	120	55	1
Where<br>$Frequence=\sum_{i\neq j}^{m}E_{ij}$	*T*	1	30	10	1
Post-processing	*d* (km)	2.5	2.5	2.5	2.5

OLS – annual DMSP OLS NTL products;

DNB – monthly S-NPP VIIRS DNB NTL products;

OLI – Landsat-8 OLI band6 images;

PALSAR – ALOS-1 PALSAR HH polarization mosaic data

n – the number of time-series satellite images/products covering the same area;

MS – mask used in the order statistic filtering (OSF);

MR – radius of the disk mask used in the OSF;

r^{th} – the rth element of the local image corresponding to the sliding mask;

Area – area of detected sea surface targets was used to set potential platforms apart from chunk of clouds;

m – the number of pair-wise comparison, depending on the number of time-series satellite images;

T – is used to segment offshore platforms from other noises according to their occurrence frequency;

d – distance of the detected targets to the coastlines.

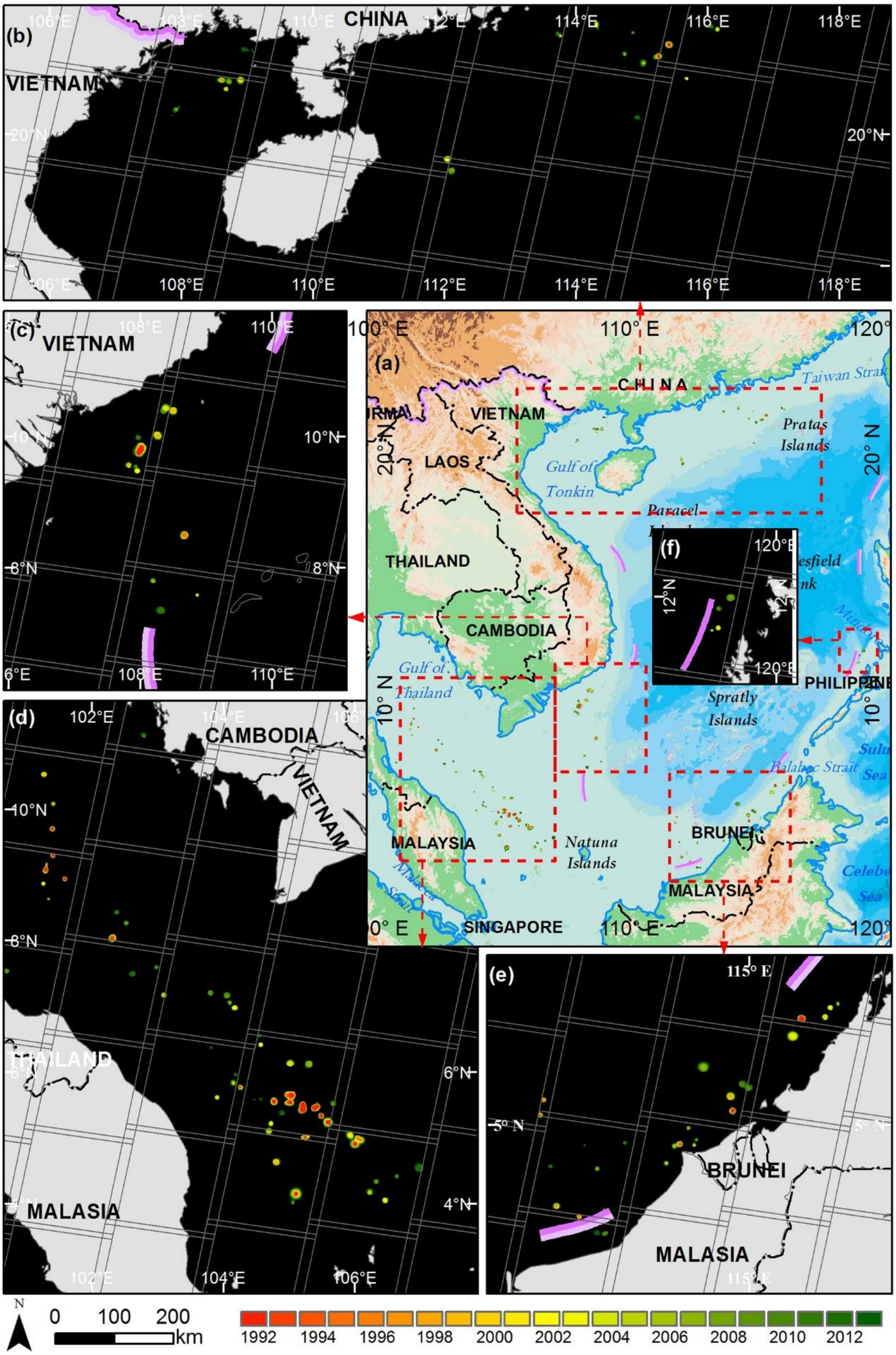

Supplementary Figure 1 | **Accumulation of persistent flaring regions extracted from the yearly DMSP OLS Nighttime Light products** (from 1992 to 2013). (a) The whole of the SCS; (b) Gulf of Tonkin and offshore of the Pearl River mouth; (c) the southeast coast offshore of the Indo-China Peninsula; (d) from the Gulf of Thailand to the west of the Natuna Islands; (e) the north coast offshore of Kalimantan Island; (f) offshore coasts of the Philippine Islands. The map data was made with Natural Earth (http://www.naturalearthdata.com/). The figure was generated by Y.L. using ArcMap 10.0 (http://www.esrichina.com.cn/).

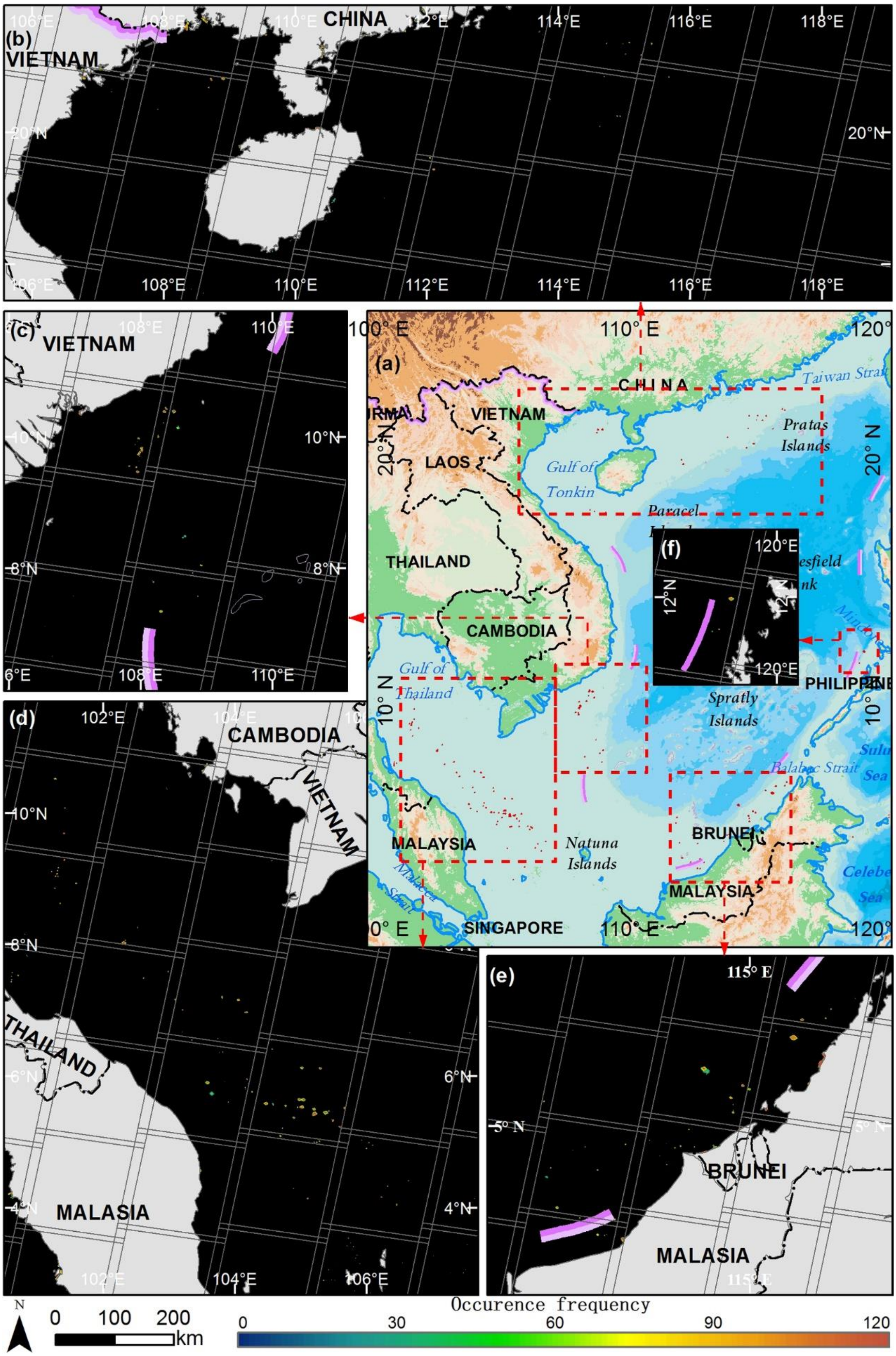

Supplementary Figure 2 | **Occurrence frequency of persistent flaming regions extracted from the monthly S-NPP VIIRS DNB Nighttime Light products** (from Jan, 2014 to May, 2015). (a) The whole of the SCS; (b) Gulf of Tonkin and offshore of the Pearl River mouth; (c) the southeast coast offshore of the Indo-China Peninsula; (d) from the Gulf of Thailand to the west of the Natuna Islands; (e) the north coast offshore of the Kalimantan Island; (f) offshore coasts of the Philippine Islands. The flaring regions from S-NPP are smaller than those from DMSP OLS products because of their finer resolution. The map data was made with Natural Earth (http://www.naturalearthdata.com/). The figure was generated by Y.L. using

ArcMap 10.0 (http://www.esrichina.com.cn/).

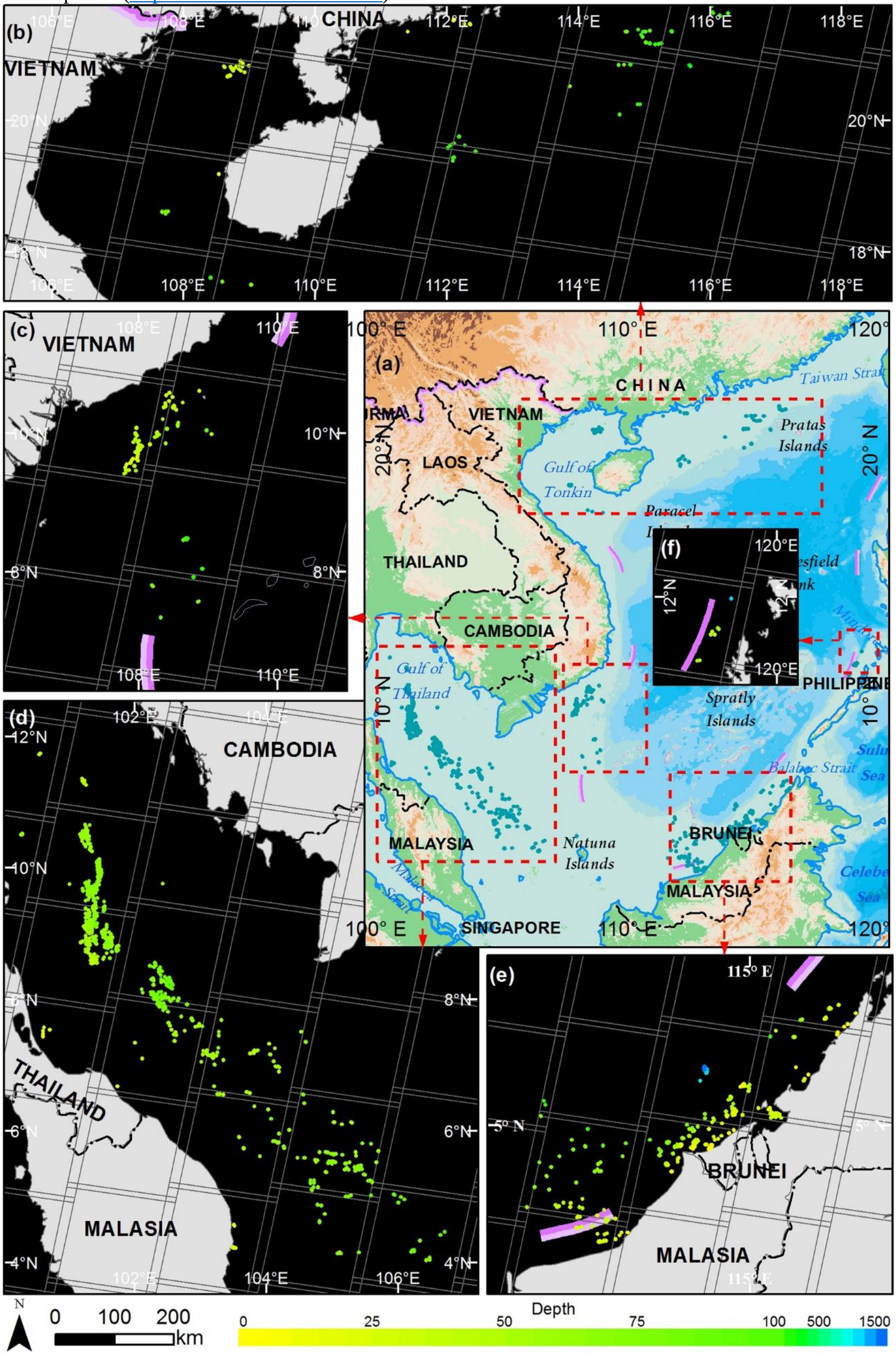

Supplementary Figure 3 | **Depth of offshore platforms in the SCS**. (a) The whole of the SCS; (b) Gulf of Tonkin and offshore of the Pearl River mouth; (c) the southeast coast offshore of the Indo-China Peninsula; (d) from the Gulf of Thailand to the west of the Natuna Islands; (e) the north coast offshore of Kalimantan Island; (f) offshore coasts of the Philippine Islands. The figure shows that the majority of oil and gas exploitation in the SCS remains in shallow water. The map data was made with Natural Earth (http://www.naturalearthdata.com/). The figure was generated by Y.L. using ArcMap 10.0

(http://www.esrichina.com.cn/).

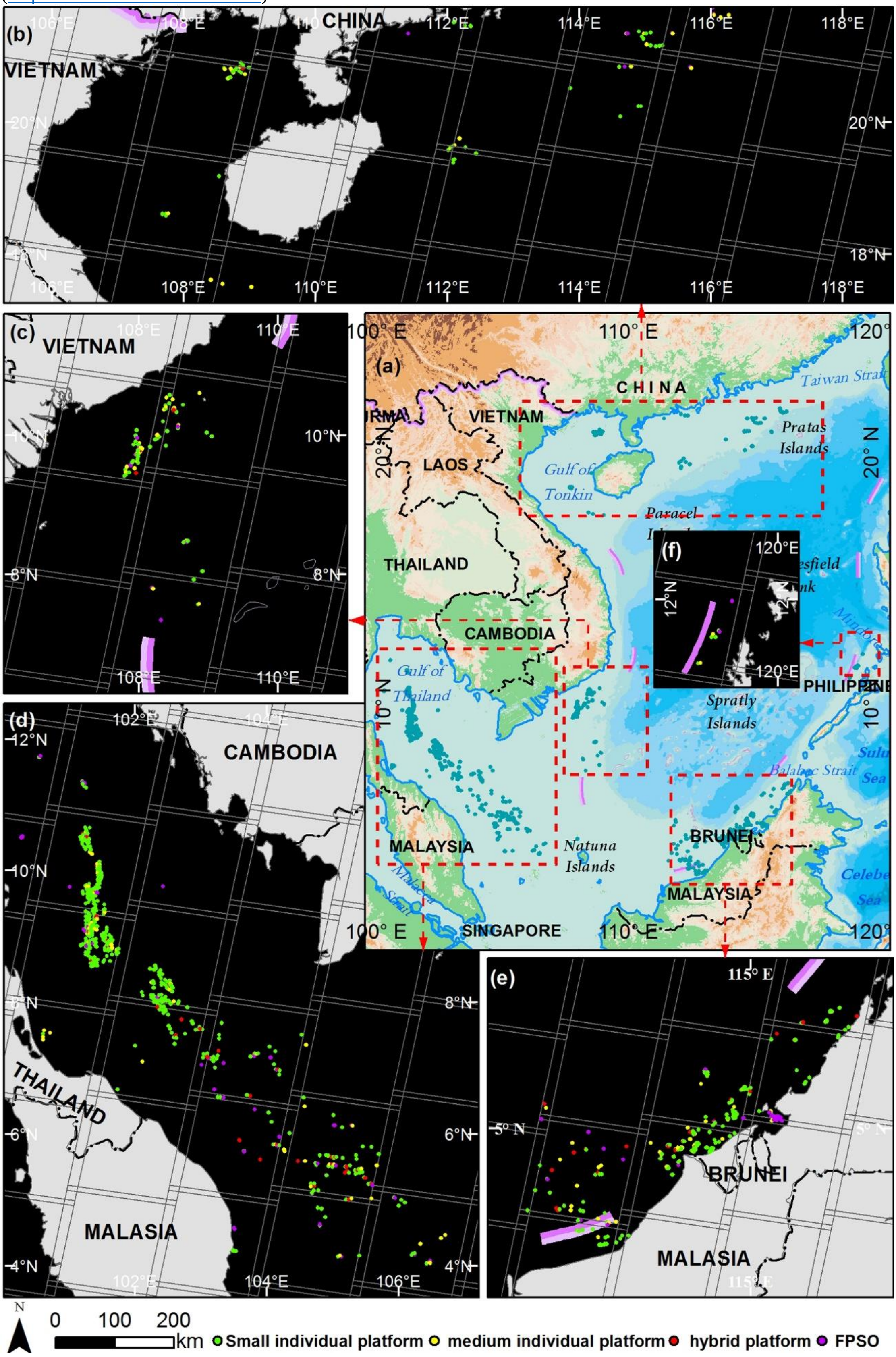

Supplementary Figure 4 | **Type of offshore platforms in the SCS**. According to the Landsat-8 OLI panchromatic images (15 m resolution) with high quality, the platforms were classified into four categories: small individual platforms (≤ 4 pixels), medium individual platforms (> 4 pixels), hybrid platforms connected by several sub-platforms, and FPSO exhibiting a ship-shape. The map data was made with Natural Earth (http://www.naturalearthdata.com/). The figure was generated by Y.L. using ArcMap 10.0 (http://www.esrichina.com.cn/).

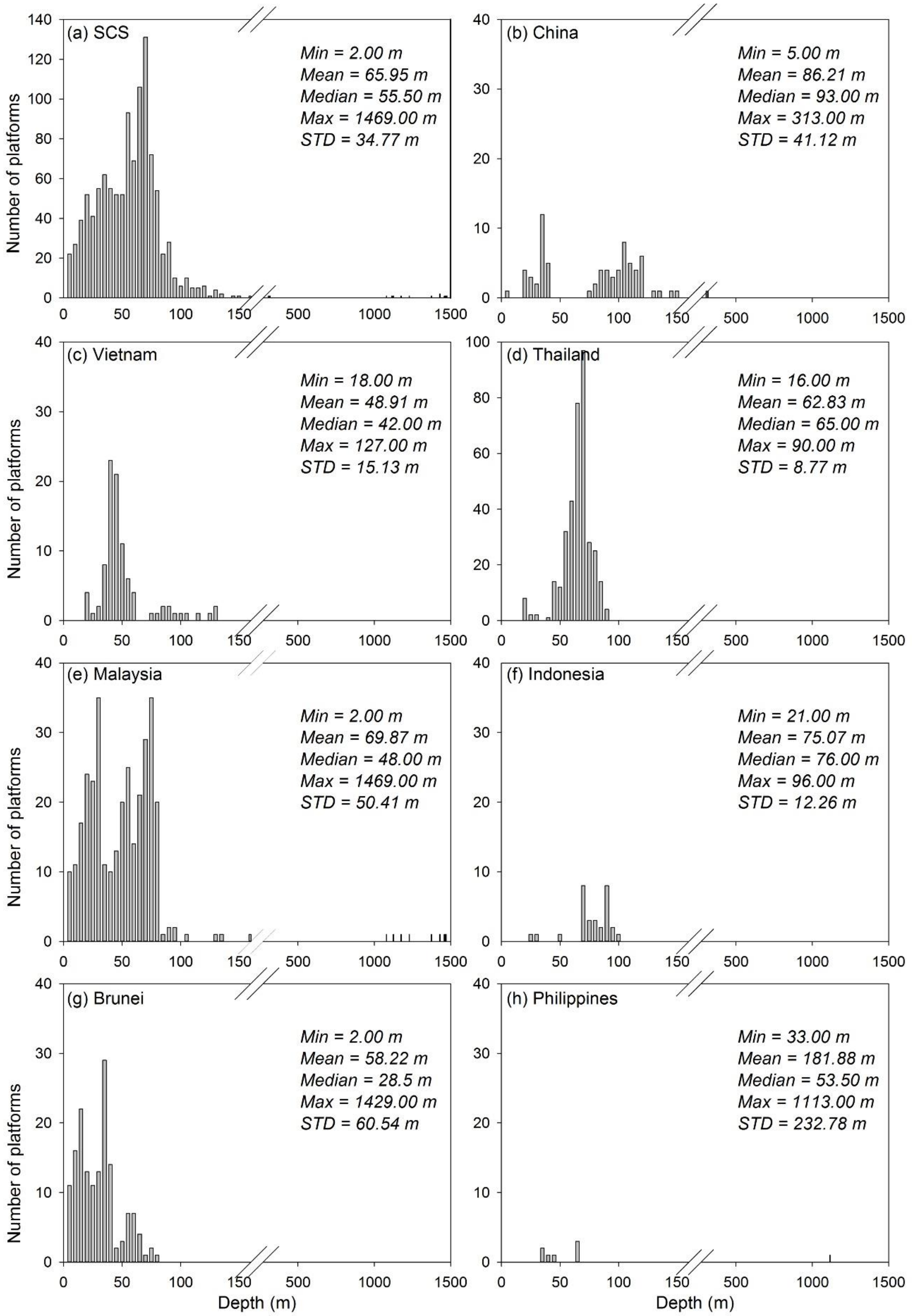

Supplementary Figure 5 | **Depth of offshore platforms in the SCS.** (a) the whole of the SCS; (b) China; (c) Vietnam; (d) Thailand; (e) Malaysia; (f) Indonesia; (g) Brunei; and (h) Philippines. Note that offshore platforms in the Malaysian-Thai Joint Development Area (MTJDA), and Malaysian-Vietnamese Joint Economic Development Zone (MVJEDZ) were not counted. The figure was generated by Y.L. using SigmaPlot 12.5 (http://www.sigmaplot.com/).

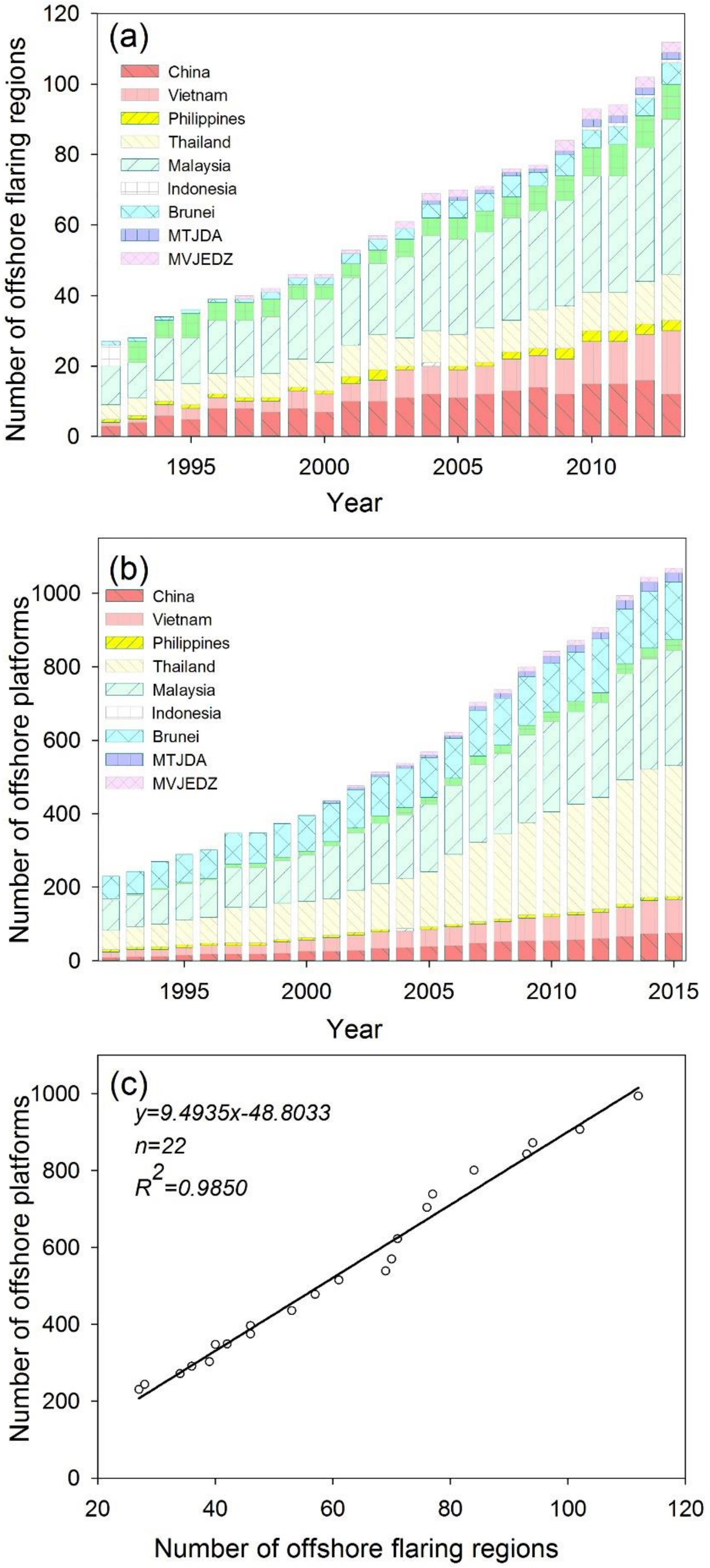

Supplementary Figure 6 | **Annual number of offshore flaring regions and offshore platforms in the SCS.** (a) The annual number of persistent flaring regions extracted from yearly DMSP OLS NTL products during 1992-2013; (b) the annual number of offshore platforms extracted from archived moderate images taken during 1992-2015; and (c) the strong linear relationship between the annual number of persistent flaring regions and the annual number of offshore platforms extracted from archived moderate resolution images suggests that the NTL products are effective indicators for offshore platforms detection. The figure was generated by Y.L. J.S., and W.C. using SigmaPlot 12.5 (http://www.sigmaplot.com/).

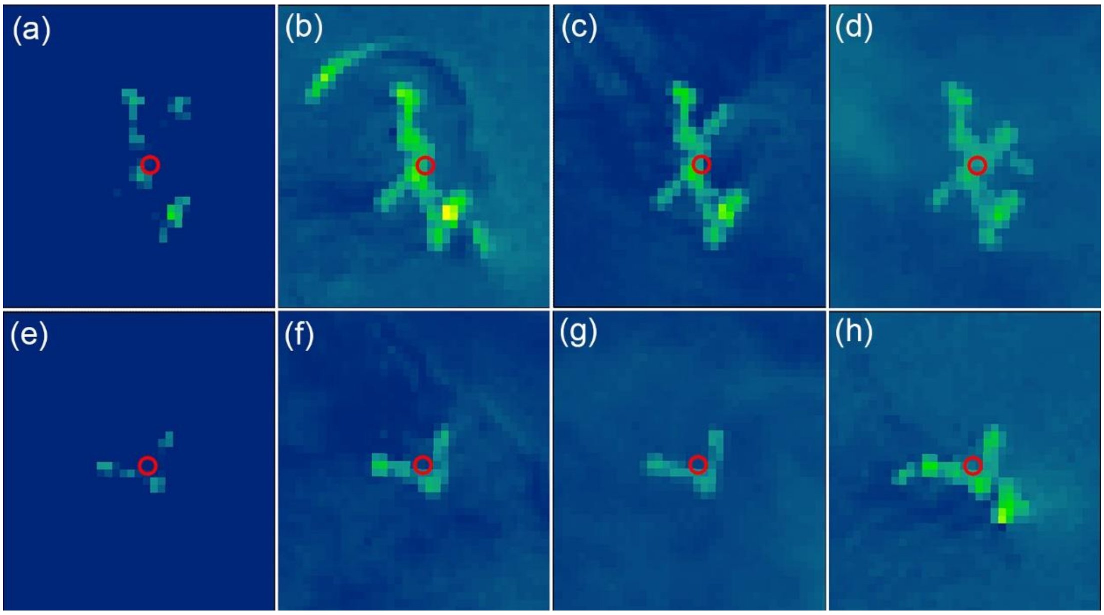

Supplementary Figure 7 | **The expansion procession of two hybrid platforms exhibiting in the short-wave infrared (SWIR1, Band6) of Landsat-8 OLI images acquired in different period.** The Landsat-8 OLI SWIR data were available from the Global Visualization Viewer of the U.S. Geological Survey (http://glovis.usgs.gov). The figure was generated by Y.L. using ArcMap 10.0 (http://www.esrichina.com.cn/).

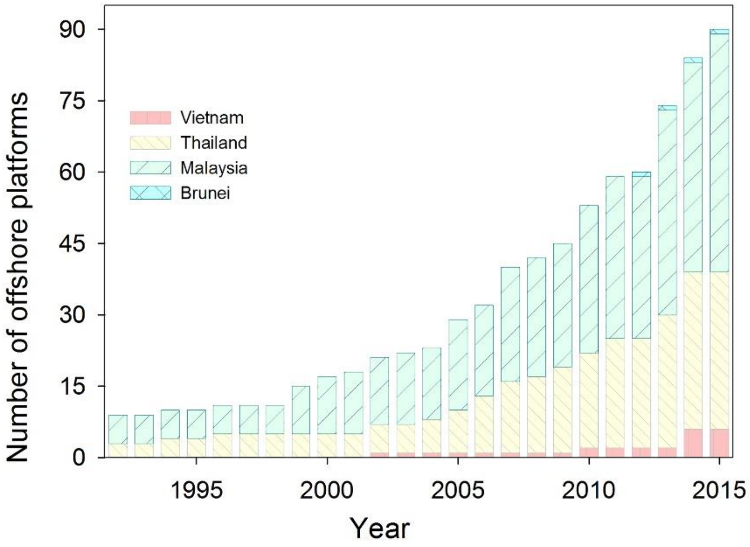

Supplementary Figure 8 | **Annual number of offshore platforms locating in the overlapping maritime area in the SCS.** The figure was generated by Y.L. using SigmaPlot 12.5 (http://www.sigmaplot.com/).

Supplementary: the South China Sea

The South China Sea (SCS) is crowded, environmentally sensitive and strategically important. It is the third largest marginal sea in the world, bordered by the Chinese mainland and Taiwan to the north, the Philippines to the east, Vietnam, Thailand, and Cambodia to the west, and Brunei, Singapore, Indonesia, and Malaysia to the south (Supplementary Fig. S9). Although it is ecologically a distinct large marine ecosystem, the Gulf of Thailand is often included in the reference made to the SCS. The SCS has an area of 3.3 million km^2 excluding the Gulfs of Thailand and Tonkin, and up to 3.8 million km^2 if these gulfs are included[1]. The SCS commingles with the Pacific Ocean via the Luzon Strait and Taiwan Strait in the northeast, and with the Indian Ocean through the Malacca Strait in the southwest. Thus, the SCS has become a vital sea-lane through which one third of world trade passes. In this paper, the study area is defined between 0-22°N and 99-124°E, including the Gulf of Thailand and the Strait of Malacca.

In terms of oil and gas resources reserves in the SCS, there are no exact numbers because of the complex geological conditions, low degree of exploration, and maritime disputes[2, 3, 4]. The U.S. Energy Information Administration estimates the SCS contains approximately 11 billion barrels of oil and 190 trillion cubic feet (TCF) of natural gas in proved and probable reserves[5]. Although various parties hold disparate estimates of the reserves, there is no doubt the SCS is endowed with major oil and gas resources, being one of the world's four big oil and gas maritime areas. An overwhelming majority of these hydrocarbon resources are distributed in offshore sedimentary basins, including the Pearl River mouth basin, Song Hong basin, Phu Khanh basin, Cuu Long basin, Nam Con Son basin, South China Sea Platform, Palawan Shelf basin, Greater Sarawak basin, Brunei Sabah basin, and Malay basin [6]. Almost all of these hydrocarbon basins are either wholly or partially subject to competing maritime claims (the exception being the Pearl River Mouth Basin which is claimed only by China)[2].

The abundant oil and gas resources in the SCS have been the focus of littoral states since 1960. Prior to the 1950s, most of the oil exploration were done onshore until some preliminary evidence of oil deposits were found in the offshore areas of Peninsular Malaysia and the discovery of the Seria Oilfield off the coast of Brunei[7, 8]. In 1962, oil was discovered offshore of Sarawak (Malaysia). The first offshore oilfield to begin operation was the West Lutong oilfield in Sarawak in 1968, and offshore platforms in the SCS have increased continuously during the past forty years. The subsequent issues, including maritime disputes, transboundary pollution, offshore decommissioning, etc., result in an increasing requirement to improve knowledge of the amount and distribution of offshore platforms in the area, for marine environment protection, crisis management, and foreign policy formulation.

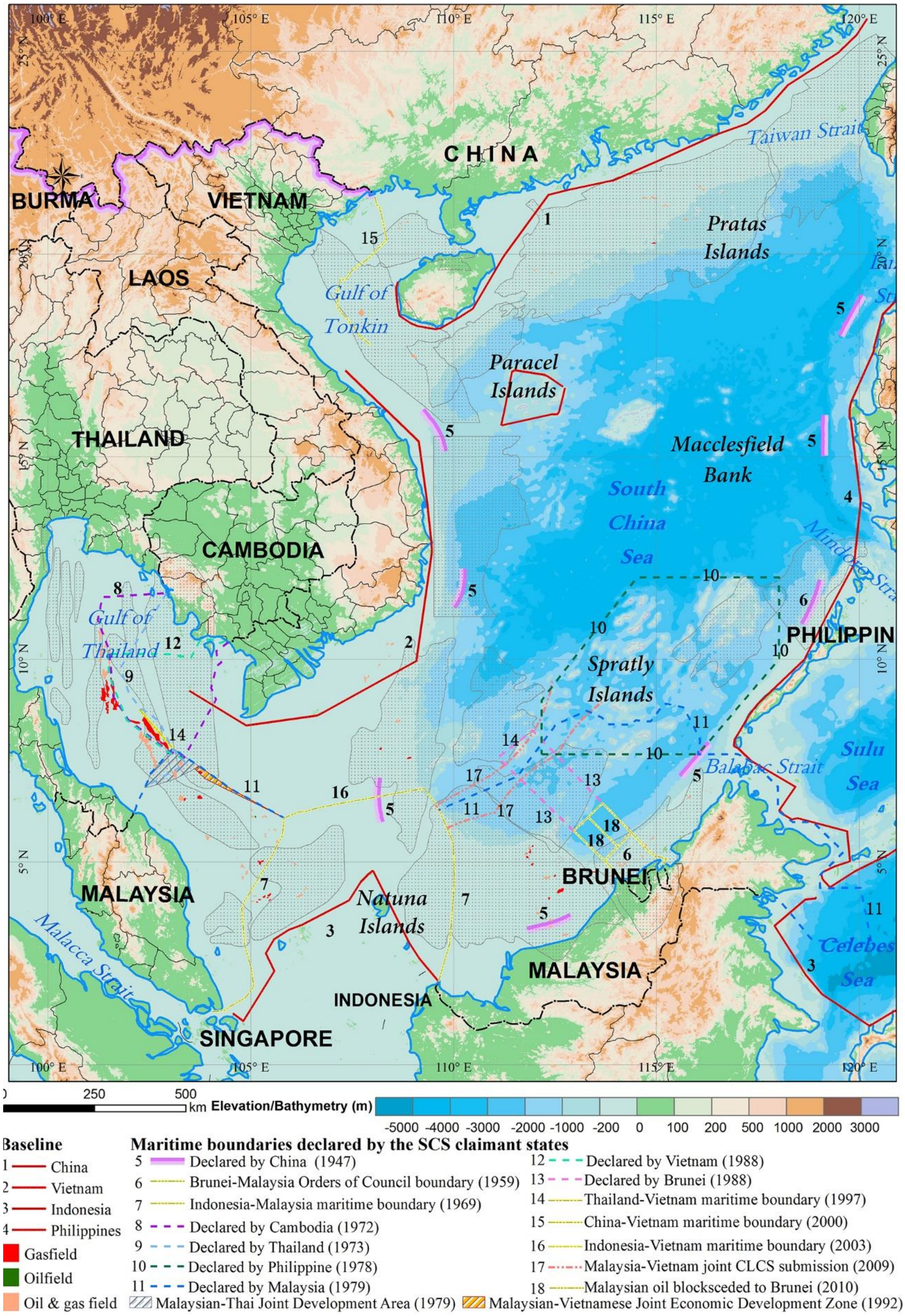

Supplementary Figure 9 | **Distribution of oil and gas basins and maritime boundaries declared by the SCS claimant.** Note that Vietnam claims the Paracel and Spratley Islands, and Philippines claims some of the Spratley Islands. However, the extent of their claim has not been delimited. The map data was made with Natural Earth (http://www.naturalearthdata.com/). The figure was generated by Y.L. using ArcMap 10.0 (http://www.esrichina.com.cn/).

Supplementary: data sets

(i) Gridded bathymetric data. Bathymetric data of the SCS (grid size: 30 arc-second) was collected from the British Oceanographic Data Centre (BODC, http://www.bodc.ac.uk/data/online_delivery/gebco/, accessed: October 11, 2012).

(ii) Shoreline and administrative areas. Shoreline and administrative areas were provided by Global Administrative Areas (http://www.gadm.org, accessed: May 10, 2012). The smaller islands, atolls, submerged reefs and banks (hereinafter referred to as reefs) were digitized manually from TIANDITU (http://www.tianditu.cn/about/index.htm, accessed: November 10, 2012).

(iii) Satellite data. Three scales of satellite data were used in this study, which were organized as low-resolution NTL products (>150 m, including DMSP OLS NTL products and S-NPP VIIRS NTL products), moderate resolution images (10-150 m, including Landsat-8 OLI images, Landsat-4/5 TM/ETM+ images, ALOS PALSAR images, ENVISAT ASAR WSM images, and Sentinel-1 images), and high resolution images (<5 m, including GF-1 and ZY-3 images). Supplementary Table 1 is a detailed summary of the satellite data used in this study. The spatio-temporal coverage of the used satellite images is shown in Supplementary Fig. S10.

(a) *DMSP OLS NTL products (1992-2013).* A total of 34 yearly DMSP OLS NTL products (Version 4, 1992-2013) were downloaded from National Oceanic and Atmospheric Administration (NOAA) National Geophysical Data Center (http://ngdc.noaa.gov/eog/), and were derived from all available archived DMSP OLS smooth resolution data for calendar years. These products excluded sunlit, glare, moonlit, and clouds. These NTL data spanning 1992-2013 were obtained by six individual sensors: F10 (1992-1994), F12 (1994-1999), F14 (1997-2003), F15 (2000-2007), F16 (2004-2009) and F18 (2010-2013). These products provide global latitude-longitude grid having a resolution of 30 arc seconds with a 6-bit digital number (DN) ranged from 0 (background) to 63 (saturated)[18].

(b) *S-NPP VIIRS NTL products (1992-2013).* A total of 16 monthly S-NPP VIIRS DNB products (Version 1, 15 arc seconds resolution, from January 2014 to May 2015) were also downloaded from NOAA's National Geophysical Data Center. The products provide monthly average radiance composite images using the VIIRS DNB nighttime data, which is filtered to exclude data impacted by stray light, lightning, lunar illumination, and cloud-cover[19].

(c) *Landsat-8 OLI images.* A total of 1035 Landsat-8 OLI images taken during 2013-2015 were downloaded through the Global Visualization Viewer of the United States Geological Survey (USGS, http://glovis.usgs.gov/). These products, covering 92 tiles, were taken from April, 2013 to March,

2015. More than eleven time-series images are contained in each tile. All of the collected Landsat-8 OLI images have a spatial resolution of 30 m (band 1-7 and 9; 15 m panchromatic band)[20, 21]. The geometric accuracy of Landsat-8 OLI L1T can reach to 12 meters (i.e., approximately a half pixel in OLI images) [22]. Moreover, the South China Sea is a tropical sea, and is often affected by clouds - hence, most of the images are of poor quality. The cloud cover ranges from 0.04 to 61.33%, with a mean of 15.49% (n = 1035 images).

(d) *Landsat-4/5 TM/ETM+ images*. A total of 1196 Landsat 4/5/7 TM and ETM+ images taken from 1991 to 2013 were obtained from USGS. All of the collected Landsat-4/5 TM/ETM+ images have a spatial resolution of 30 m (band 1-7; 15 m for ETM+ panchromatic band). The geometric accuracy of Landsat-4/5 TM/ETM+ is lower than that of OLI, approximately 150 m [23, 24, 25].

(e) *ALOS PALSAR images.* Four phases of ALOS PALSAR HH polarization mosaic data, 320 tiles (1°× 1°) for each phase, covering the whole of the SCS, acquired between June and October during 2007 and 2010[26], were downloaded from the Japan Aerospace Exploration Agency (JAXA, http://www.eorc.jaxa.jp/), to validate the extracted offshore platforms from OLI images. The mosaic images were resampled to 25 m with a geometric accuracy of no more than 7.8 m[27].

(f) *ENVISAT ASAR WSM images.* Totally 573 ENVISAT ASAR WSM images taken from 2005 to 2009 were obtained from the Satellite Remote Sensing Ground Receiving Station in Hong Kong. ENVISAT ASAR images can also be downloaded from European Space Agency (ESA, http://www.esa.int/ESA/) after permission.

(g) *Sentinel-1 images*. A total of 86 Sentinel HH polarization images taken from December, 2014 to June, 2015 were downloaded from ESA Sentinels Scientific Data Hub (https://scihub.esa.int/).

(h) *Chinese GF-1 and ZY-3 high resolution images*. A total of 46 images acquired by the Chinese GF-1 satellite and 43 images taken by the Chinese ZY-3 satellite, were obtained from the China Center for Resource Satellite Data and Applications (CRESDA, http://www.cresda.com). The GF-1 images had a panchromatic band (2 m resolution) and four multi-spectral bands (8 m resolution), covering 35 × 35 km^2 [28]. The ZY-3 images had four multi-spectral bands (6 m resolution), covering 50 × 50 km^2 [29].

(i) *Google Earth high resolution image snapshot.* Google Earth provides numerous snapshots of high-resolution images covering coastal zones. We overlapped all excluded targets from coastal zones (10 km from the coastline).

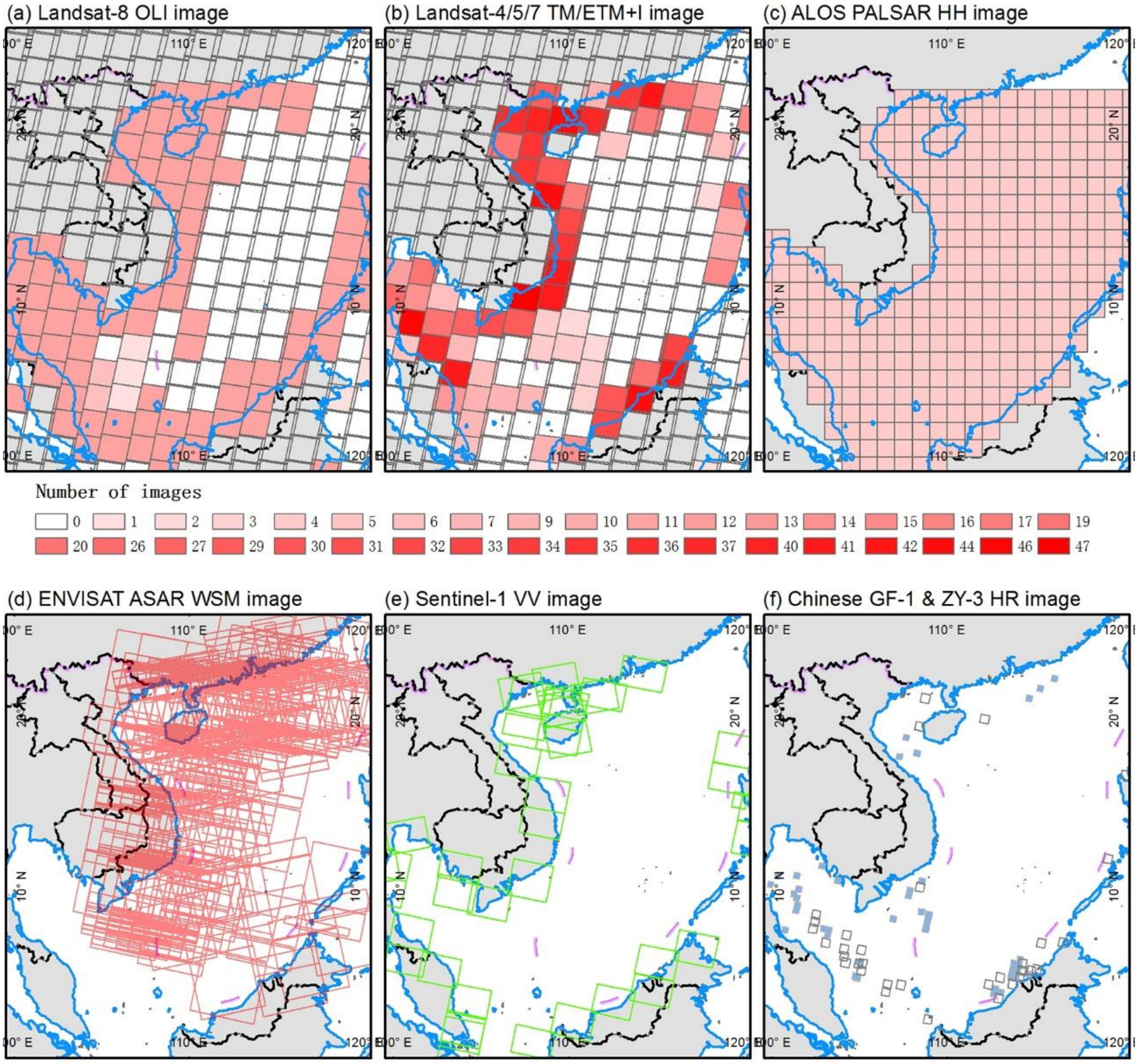

Supplementary Figure 10 | **The spatial coverage and frequency of the medium/high resolution images used for offshore platform detection and validation.** (*a*) The coverage of the collected Landsat-8 OLI images. A total of 1035 Landsat-8 OLI images taken from April, 2013 to March, 2015, were collected. Among these tiles, a total of 83 tiles have more than 11 phases' images taken from 2013 to 2015. (*b*) The coverage of the collected Landsat-4/5/7 TM/ETM+ images. A total of 1196 Landsat-4/5/7 TM/ETM+ images taken from 1991 to 2013, were collected. (*c*) The coverage of ALOS PALSAR HH polarization mosaic data. A total of 320 tiles is required to completely cover the SCS, and each tile has one image per year taken from 2007 to 2010. (*d*) A total of 573 ENVISAT ASAR WSM images, taken from 2005 to 2009, were collected. (*e*) A total of 86 Sentinel-1 VV images taken from November, 2014 to June, 2015. Each tile has two images acquired with a least a three-month span. (*f*) 89 Chinese GF-1 and ZY-3 high resolution images. The map data was made with Natural Earth (http://www.naturalearthdata.com/). The figure was generated by Y.L. and J.S. using ArcMap 10.0 (http://www.esrichina.com.cn/).

Supplementary: Method for offshore platform detection

Offshore platforms are essential infrastructure required to drill wells, extract, process, and temporarily store crude oil and natural gas. In the SCS, there are various types of offshore platforms, including the old model of small and fixed jackets in shallow coastal waters, large floating production storage and offloading units (FPSO) in deep water, jack-up, semi-submersible production units, stacked-led structures, spars, and others. Depending on the offshore oil/gas field water-depth and situation, platforms can be fixed to the ocean floor, or be moored. According to our previous study, the shift of position of a FPSO is no more than tens of meters[30]. In the SCS, most platforms are small and fixed. Usually, these platforms exhibit no more than 10 pixels in a 30 m resolution images, while the size of some hybrid platforms which are connected by several sub-platforms can reach dozens of pixels.

In general, the offshore oil and gas platforms are generally made of various grades of steel, from mild steel to high-strength steel, although some of the older structures were made of reinforced concrete. These metallic structures with a high degree of exposure to sunlight usually exhibit a high digital number (DN) in the short-wave infrared band of the optical images, and high radar cross section (RCS) in SAR images. In addition, some platforms burn off excess associated petroleum gas (APG), which make them rather significant in the nighttime.

Although offshore platforms may be the most significant manmade structures in the sea, detecting them and determining their attribute from space-borne images is challenging. The problems include the following (Supplementary Fig. S11): *(1) Small target*. The detection of offshore platforms which usually exhibit one or several pixels in a moderate resolution images without sufficient supporting information (e.g., shape, structure, and etc.) is difficult. *(2) Dim target*. Many small platforms also present an inconspicuous difference compared with their surroundings, especially for optical images. (*3*) *Noisy backgrounds*. The quality of satellite images suffers from poor imaging conditions (e.g., clouds, mists), underlying surface (e.g., waves, ripples, turbidity), and the imaging device (e.g., stripe-missing, speckle noise); thus, offshore platforms are usually immerse in heavy noise. *(4) Numerous false alarms*. Offshore platform detection also suffers from numerous vessels which also present similar features. *(5) Vast sea area*. To pick out platforms over a vast sea area (e.g., the SCS) from satellite images (usually low signal-noise-ratio), automation and robustness need to be considered. To extract offshore platforms (usually tiny and dim) over a vast sea area (usually having a high noise and clutter background) from satellite images (usually influenced by poor weather), we improve the automated method for extracting offshore platforms (AMEOP) from time-series OLI images which we proposed previously[30], and the following general technical framework was used.

(1) Sea surface target detection from mono-temporal satellite images according to spot-like and sparse principles. We assume the signature of sea surface targets in the satellite image is composed of background, sea surface targets (including offshore platforms, vessels, clouds, and others), and noise (Eq. 1). Hence, the signature of a sea surface target at t moment can be denoted by Eq. 2.

$$f(x,y,t)= f_s(x,y,t)+ f_b(x,y,t)+ f_n(x,y,t) \quad (1) \qquad f_s(x,y,t)= f(x,y,t)- f_b(x,y,t)- f_n(x,y,t) \quad (2)$$

Where, $f(x, y, t)$ is the DN of pixel (x, y) at t moment, while $f_s(x, y, t)$, $f_b(x, y, t)$, and $f_n(x, y, t)$ are DN of sea surface target, background, and noise, respectively.

In practice, an order-statistic filtering (OSF) was used to approximate the background (Eq. 3). Specifically, the pixel in a sliding disk-like window were ascending sorted according to their values, and the rth element was selected to be the background value of the domain. In general, a higher order (r) will be capable of better suppressing non-uniform and complex backgrounds, since offshore platforms are (*i*) spot-like, (*ii*) have a higher DN value than those of their surroundings pixels, (*iii*) are tiny, and (*iv*) sparsely distributed. Note that the order (r) varies for different scales of the satellite images used: the order (r) for moderate resolution optical images should be set higher to resist the influence of the ripples generated by waves or currents (Supplementary Fig. S11i and k), while the order (r) for the low resolution NTL products is relatively lower because the flaring of platforms often exhibit halos that are much larger than their actual size (Supplementary Fig. S11b, c, and d). As a general rule, noise sources in satellite images are unknown. Hereafter, the local standard deviation of the sliding window is used to approximate the noise. However, the standard deviation is usually subtle for a homogeneous background, especially using a large sliding window where the difference has been unexpectedly diluted, hence, a user-defined constant (ζ) is added to better suppress those noises in the smooth regions (Eq. 4).

$$f_b(x,y,t_i) \approx r^{th}\text{-}\underset{u,v}{OSF}\left(f\left(x-u, y-v, t_i\right) + w\left(u,v,t_i\right)\right) \quad (3)$$

$$f_n(x,y,t) \approx \underset{u,v}{STD}(f(x\text{-}u,y\text{-}v,t) + w(u,v,t)) + \xi \quad (4)$$

(2) Noise removal and offshore platform detection from time-series satellite images according to the position invariance principle. Numerous false alarms, including vessels, pulse noises, and others, will be unexpectedly detected in the above process from mono-temporal satellite images. For two pairwise satellite images with high geometric accuracy, the position-invariance principle can be applied to discriminate between offshore platforms and moving vessels or random small clouds. In many cases, the over-density noise may not be completely removed by pairwise comparison. Moreover, optical images are highly

susceptible to clouds, and thus offshore platforms may be covered by prevailing clouds in some specific phases' optical images. Subsequently, a time-series-image strategy was used to robustly eliminate these remnant errors (Eq. 5). In the accumulated image of coupled comparisons, offshore platforms usually had a higher occurrence frequency (high position-invariant consistency in time-series images) than the residual and randomly-distributed small clouds.

$$Frequency = \sum_{i \neq j}^{n} \mathrm{E}\left(x, y, t_i\right) \cap \mathrm{E}\left(x, y, t_j\right), \text{ where } \mathrm{E}\left(x, y, t_i\right) = \begin{cases} 1, IF \;\; f_s(x,y,t_i) > 0 \\ 0, Otherwise \end{cases} \quad (5)$$

(3) Self-consistency check by time-series Landsat-8 OLI images. All targets identified by the aforementioned general platform detection method were overlapped on the time-series OLI images with high quality. Through careful visual examination, we excluded mis-extracted targets, such as small islands and clouds according to their varying size/shape. The detailed extraction process for various sensors is shown in Supplementary Fig. S12-16. Supplementary Fig. S17 give examples for determining types of offshore platforms from Landsat-8 OLI images. Supplementary Table S3 lists all detailed parameters for automated extraction from the used time-series images.

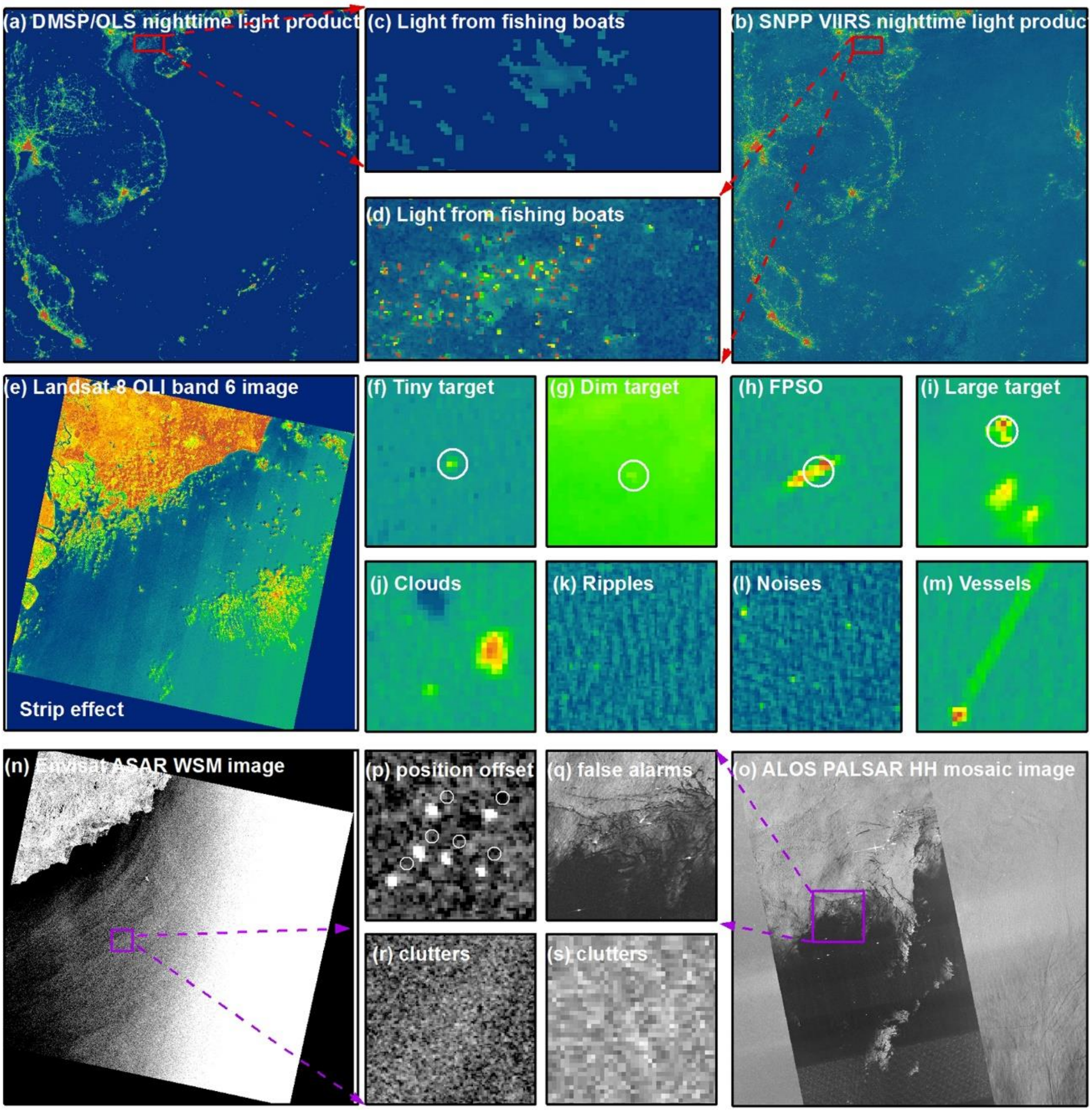

Supplementary Figure 11 | **Challenges in offshore platforms detection from space-borne images.** For NTL products, e.g., DMSP OLS products (a), and S-NPP VIIRS DNB products (b), the problem is how to robustly distinguish gas flaring/lights of offshore platforms from those of numerous fishing boats (c and d). For optical images, e.g., Landsat-8 OLI band-6 images, the automation and robustness of platform extraction are challenged by the following factors: (e) the strip effect of the images, (f) tiny targets, (g) dim targets influenced by mist, (j) false alarms including clouds, (k) ripples, (l) noises, and (m) vessels. It should be noted that high-density clouds are prevalent in the SCS, e.g. sub-figure (e) (path/row: 124/053, January 30, 2014, the cloud cover ratio is 3.61 %). Usually such optical images with a cloud cover ratio of less than 5% are rare for the SCS. The coarse geo-location accuracy of Landsat-4/5 TM images in the sea (approximately 150 m) is another issue. Although the radar cross-section of man-made targets (e.g., offshore platforms and ships) is much higher because of the effect of multiple incoming radar waves from the targets' superstructure from the flat and calm sea surface, detecting platforms from SAR images suffers from poor geo-location accuracy (p), numerous false alarms (q), and heavy background clutter (r and s). Moreover, the intensity of backscatter signal may differ with the incident angles, e.g., subfigure (n) taken by ENVISAT ASAR in WSM mode (December 19, 2008), which means that it is difficult to effectively distinguish platforms using a fixed/global threshold. The DMSP OLS NTL data was downloaded from the NGDC Earth Observation Group (EOG) of NOAA (http://ngdc.noaa.gov/eog/download.html). The Landsat-8 OLI SWIR data were available from the Global Visualization Viewer of the U.S. Geological Survey (http://glovis.usgs.gov). The ALOS PALSAR Standard Products ((c) JAXA/METI) were downloaded from the Japan Aerospace Exploration Agency (JAXA, http://www.eorc.jaxa.jp/). The figure was generated by Y.L. using ArcMap 10.0 (http://www.esrichina.com.cn/).

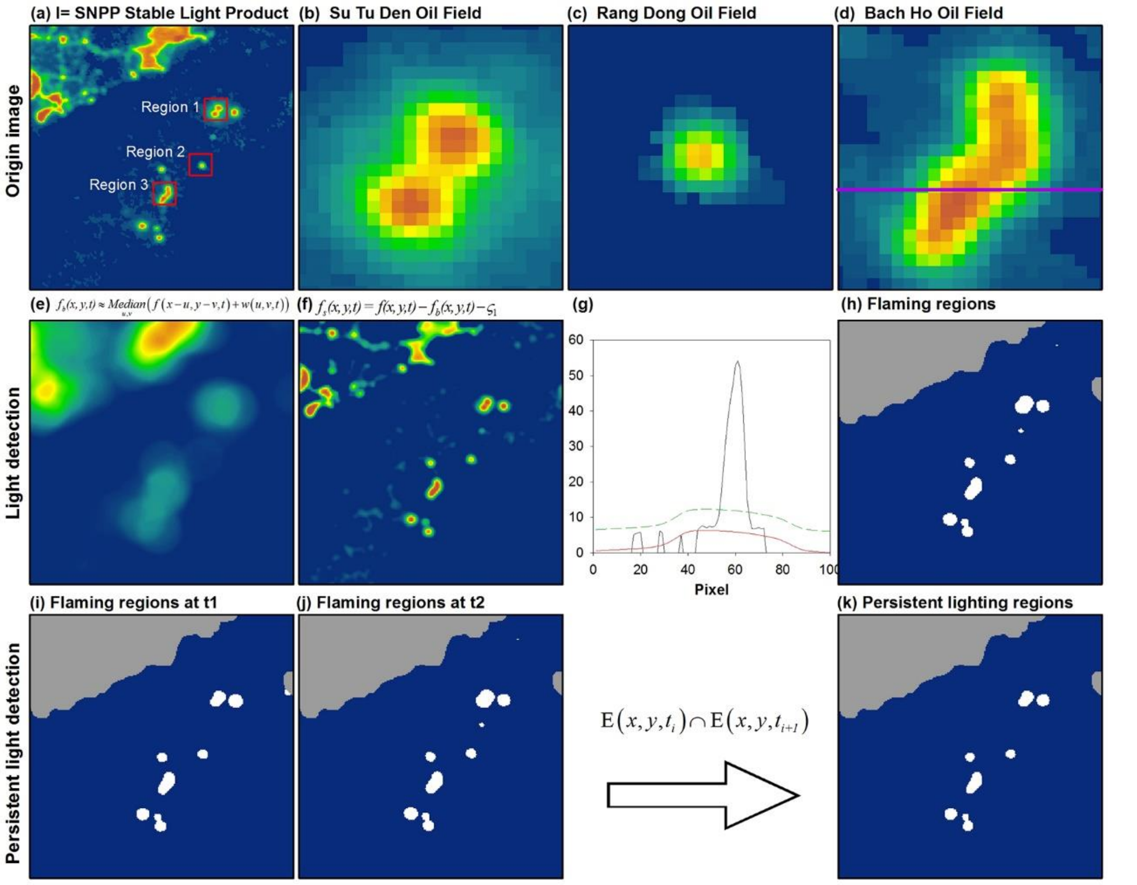

Supplementary Figure 12 | **Automatic detection of persistent light region from annual DMSP/OLS NTL product.** (*a*) Major oil fields in the Cuu Long basin offshore of the southern coast of Vietnam documented in the yearly DMSP OLS NTL data (2013, Browner = higher); (*b-d*) Su Tu Den (Black Lion), Rang Dong (Dawn), and BACH HO (White Tiger) Oil Field; (*e*) median filtering result based on a disk filter (radius = 20); (*f*) difference map between the origin image and the sum of background (assumed to be estimated by the median filtering result) and noise (assumed as a constant, $\zeta_1 = 6$); (*g*) nighttime light region detection along one dimension profile (purple line in sub-figure d); (*h*) nighttime light regions detected from the 2013 NTL product; and (k) persistent nighttime light region refined by pair-comparison with (*i*) light region detected at t_1 year and (*j*) light region detected at t_2 year. Note that the two parameters (median filter and ζ_1) used remained constant for all DMSP OLS annual NTL data. The DMSP OLS NTL data was downloaded from the NGDC Earth Observation Group (EOG) of NOAA (http://ngdc.noaa.gov/eog/download.html). The figure was generated by Y.L. using ArcMap 10.0 (http://www.esrichina.com.cn/).

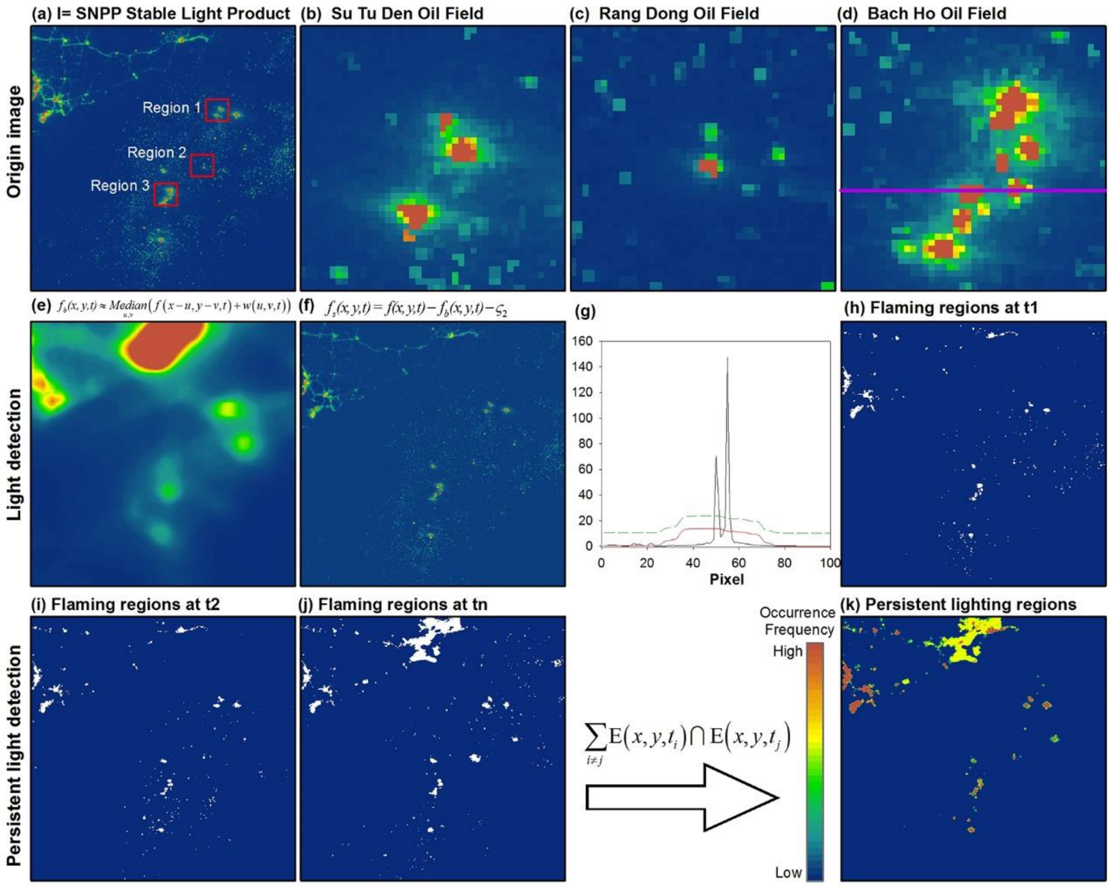

Supplementary Figure **13 | Automatic detection of persistent light region from S-NPP VIIRS monthly NTL products.** (*a*) Major oil fields in the Cuu Long basin offshore the southern coast of Vietnam documenting in the monthly S-NPP NTL data (January, 2014, Browner = higher); (*b*-*d*) Su Tu Den (Black Lion), Rang Dong (Dawn), and BACH HO (White Tiger) Oil Field; (*e*) median filtering result based on a disk filter (radius = 20); (*f*) difference map between the origin image and the sum of background (assumed to be estimated by the median filtering result) and noise (also assumed as a constant, ζ_2 = 10); (*g*) light region detection along one dimension profile (purple line in sub-figure d); (*h*) nighttime light regions detected from the May 2014 NTL product; and (k) persistent nighttime light regions during the period between January, 2014 and May, 2015 were refined by pair-comparison among 16 time-series monthly S-NPP VIIRS DNB NTL products. Note that the two parameters (median filter and ζ_2) used remained constant for all monthly S-NPP NTL data. The Suomi NPP NTL data was downloaded from the NGDC Earth Observation Group (EOG) of NOAA (http://ngdc.noaa.gov/eog/download.html). The figure was generated by Y.L. using ArcMap 10.0 (http://www.esrichina.com.cn/).

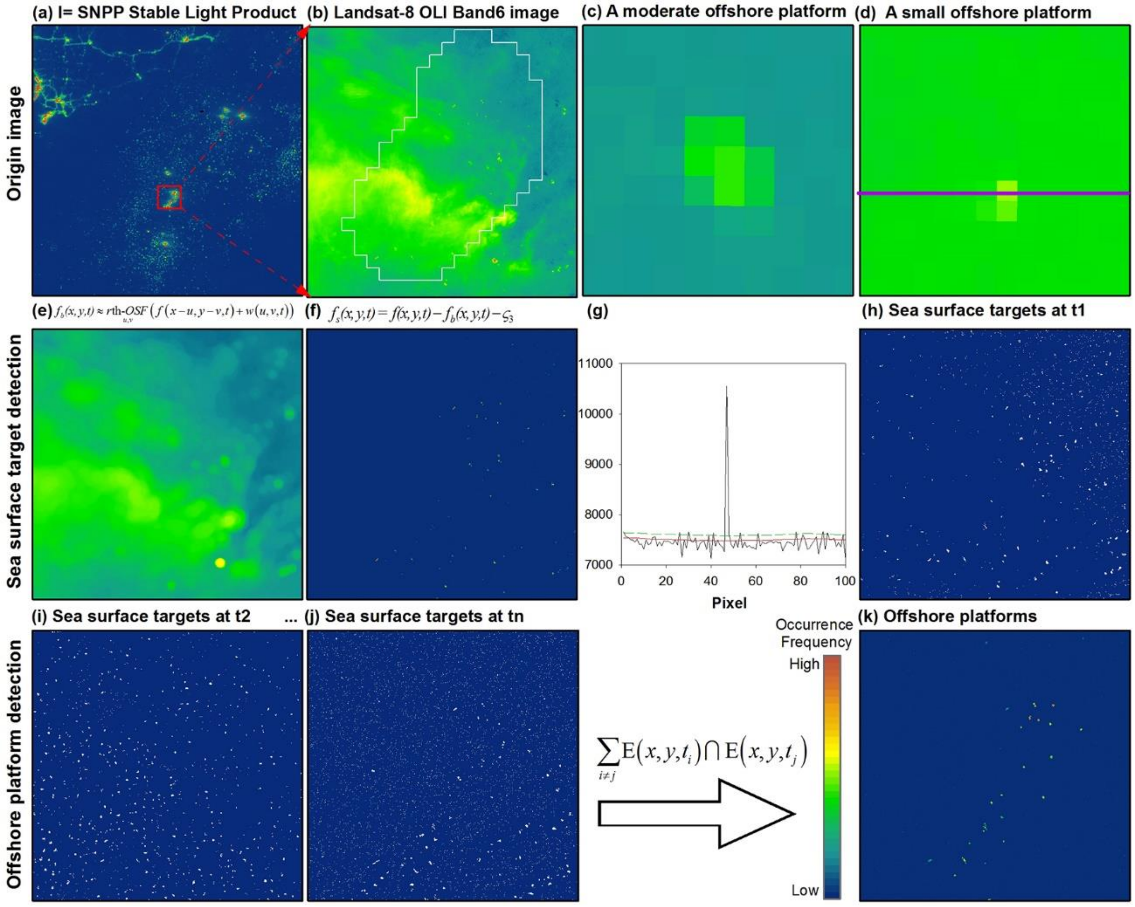

Supplementary Figure 14 | **Automatic detection of offshore platforms from time-series Landsat-8 OLI SWIR (band6) images.** (*a*) Major oil fields in the Cuu Long basin offshore of the southern coast of Vietnam documenting in the monthly S-NPP NTL data (January, 2014, Browner = higher); (*b*) the zoomed in Landsat-8 OLI Band6 image (photographed on December 29, 2013), overlain by the persistent flaring region (the white polygon) detected from NTL products. (*c*) and (*d*) a zoomed-in moderate size (eight pixels) and a small platform (two pixels) respectively, documented in the Landsat-8 OLI Band6 image. (*e*) OSF filtering result based on a disk filter (radius = 20); (*f*) difference image between the origin image and the sum of background (assumed to be estimated by the OSF filtering result) and noise (also assumed as a constant, here ζ_3 = 20 according to our experiments); (*g*) offshore platform detection along one dimension profile (purple line in sub-figure d); (*h*) sea surface targets detected from images photographed on December 29, 2013; (i) sea surface targets detected from images photographed on January 30, 2014; (j) sea surface targets detected from images photographed on February 15, 2014, and (k) occurrence-frequency generating from time-series sea surface target detected from eleven phases images taken between August 7, 2013 and January 17, 2015. Note that the two parameters (median filter and ζ_3) used remained constant for all Landsat-8 OLI Band6 images. The Landsat-8 OLI SWIR data were available from the Global Visualization Viewer of the U.S. Geological Survey (http://glovis.usgs.gov). The figure was generated by Y.L. using ArcMap 10.0 (http://www.esrichina.com.cn/).

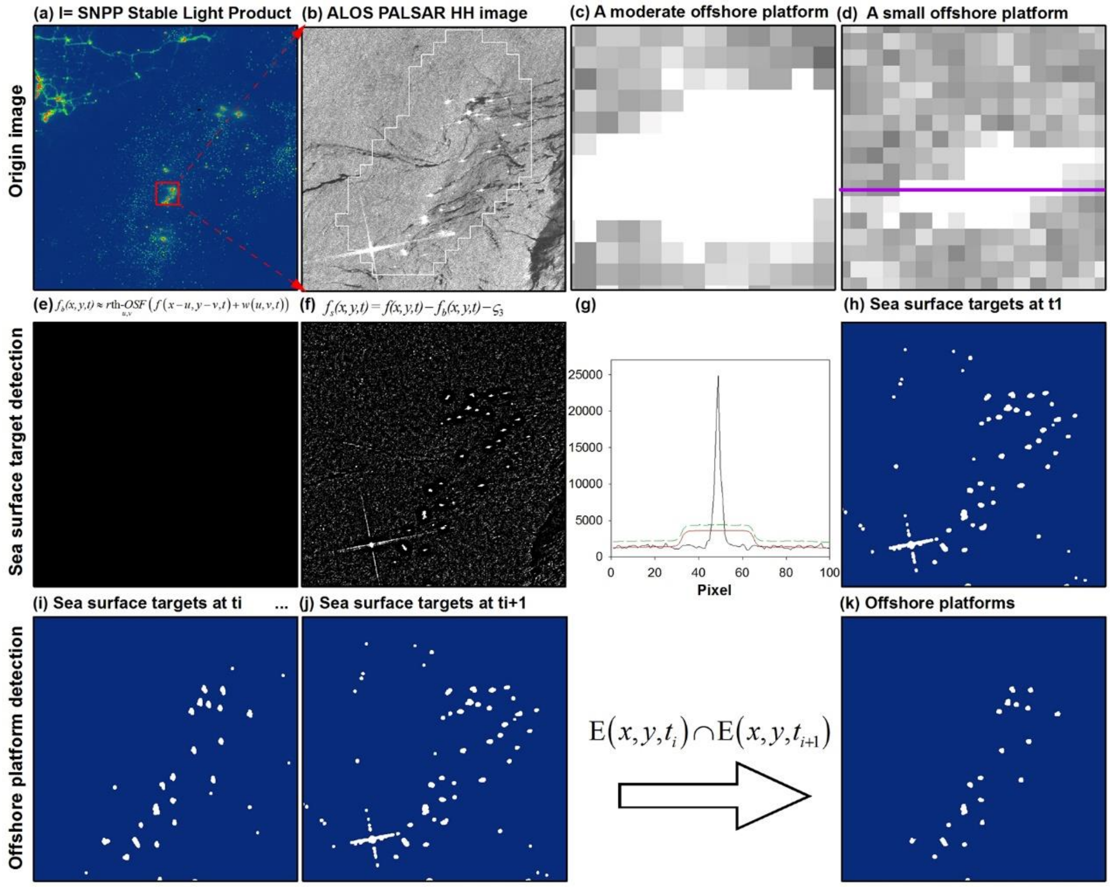

Supplementary Figure 15 | **Automatic detection of offshore platforms from two phases ALOS-1 PALSAR HH images.** (*a*) Major oil fields in the Cuu Long basin offshore of the southern coast of Vietnam documented in the monthly S-NPP NTL data (January, 2014, Browner = higher); (*b*) the zoomed in ALOS-1 PALSAR HH image; the white polygon is the persistent flaring region detection NTL products. (*c*) and (*d*) a zoomed-in moderate size and a small platform, respectively, documented in the ALOS-1 PALSAR HH image. The ALOS PALSAR Standard Products ((c) JAXA/METI) were downloaded from the Japan Aerospace Exploration Agency (JAXA, http://www.eorc.jaxa.jp/). The figure was generated by Y.L. using ArcMap 10.0 (http://www.esrichina.com.cn/).

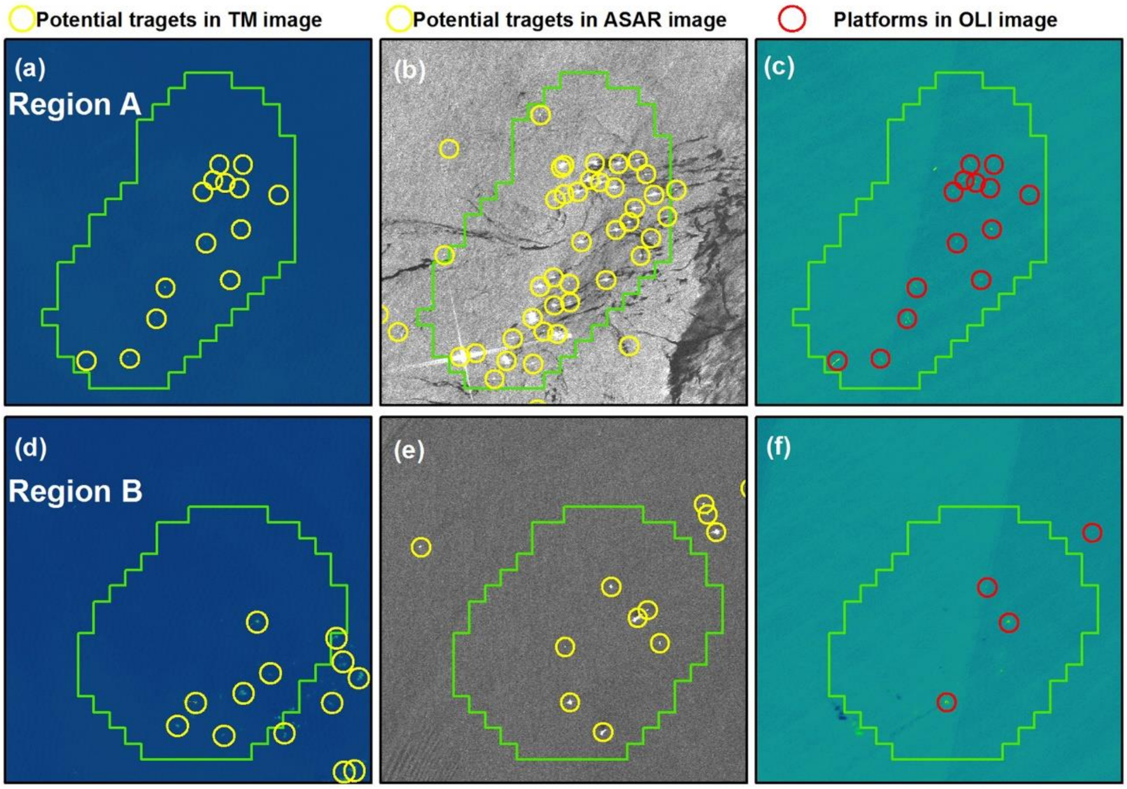

Supplementary Figure 16 | **Semi-automatic extraction of offshore platforms from archived moderate resolution images with poor geometric accuracy.** The position invariance characteristic of platforms is absolutely vital for their detection. However, the geometric accuracy of many archived satellite imageries, e.g., Landsat-4/5 TM, ENVISAT ASAR, usually reach 150 m (about 5 pixels) in the open sea, which cannot guarantee their being geographically inconsistent between pairwise images. The problem is exacerbated by the fact that few ground control points (GCPs) are available for precise geometric rectification, especially in the open sea far from land/islands. In this study, offshore platforms detected from up-to-date Landsat-8 OLI images, were used as GCPs to help geo-correct those archived images with poor geometric accuracy, and thus those platforms keeping position-invariance in the time-series geo-corrected archived satellite images were picked. (a) and (d) are two zoomed-in Band7 images acquired by Landsat-5 (path/row: 124/053), while (b) and (e) are two zoomed-in ASAR acquired by ENVISAT, The GCPs for geo-correction were carefully selected using the guidance provided by the record of persistent flaring regions (green polygons) detected from NTL products and the detected platforms, i.e., red circles in (c) and (f), from up-to-date Landsat-8 OLI images. The Landsat-4/5 TM SWIR data were available from the Global Visualization Viewer of the U.S. Geological Survey (http://glovis.usgs.gov). The ASAR images were downloaded from ESA (http://www.esa.int/ESA/). The figure was generated by Y.L. using ArcMap 10.0 (http://www.esrichina.com.cn/).

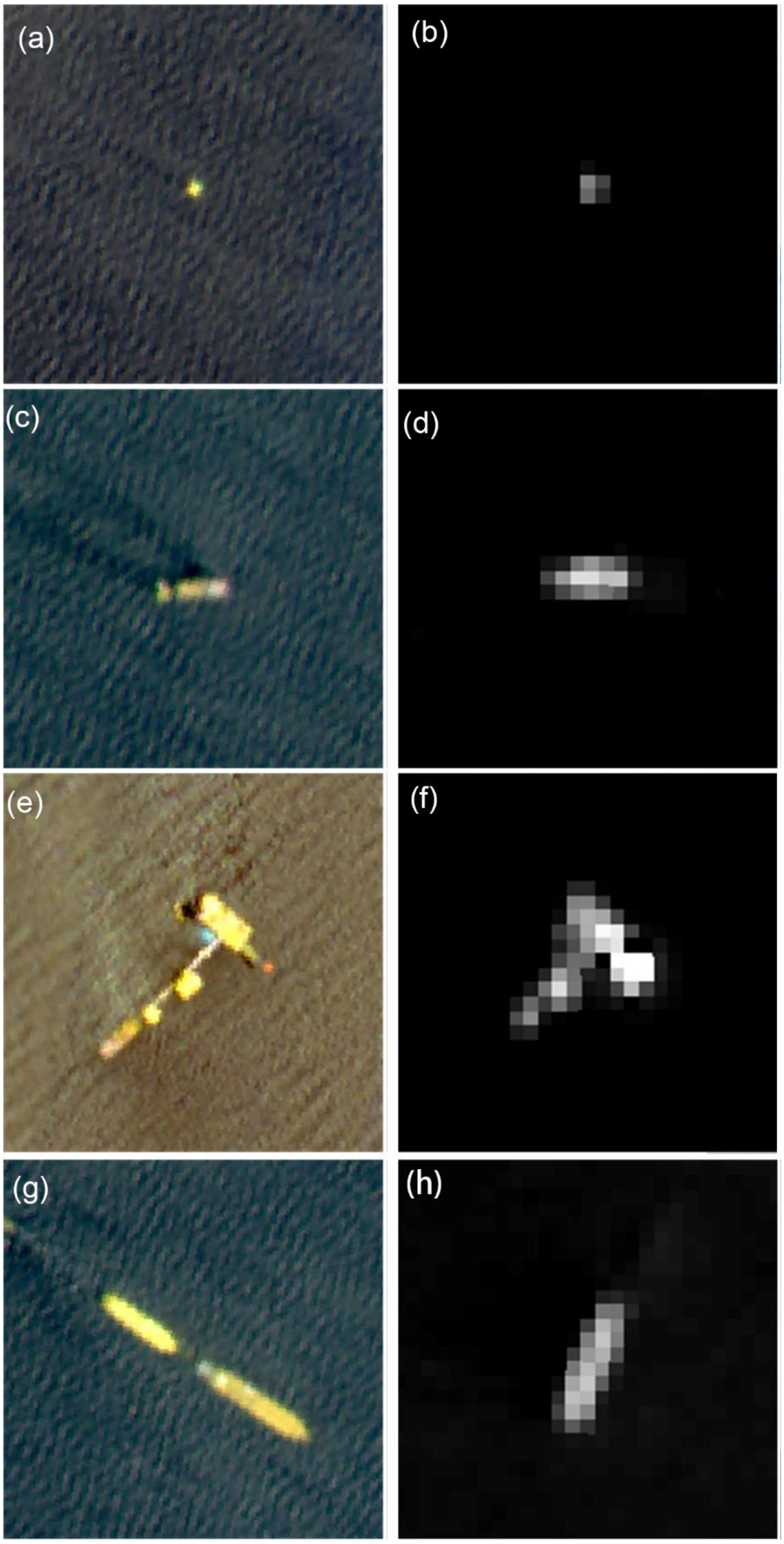

Supplementary Figure 17 | **Determining types of offshore platforms from Landsat-8 OLI images**. (a-b) a small offshore platform (approximately 10 m × 10 m) which exhibits several pixels in a Landsat-8 OLI Band6 image; (c-d) a medium size platform presenting approximately a dozen pixels; (e-f) a hybrid platform which connected by several sub-platforms showing a complex shape with dozens of pixels; (g-h) a FPSO which demonstrate a strip-shape target with dozens of pixels. The band6 data were available from the Global Visualization Viewer of the U.S. Geological Survey (http://glovis.usgs.gov). The figure was generated by Y.L. using ArcMap 10.0 (http://www.esrichina.com.cn/).

Supplementary: Validation

A total of 1311 targets with position-invariant characteristic were automatically extracted from Landsat-8 OLI images. By checking time-series OLI images with high-quality, we removed 13 errors (clouds) and 239 errors (small islands/reefs and docks/groynes, almost all located within 10 km from the coastline), with 1059 remaining. The robustness of the extractions was assessed using three capable space-borne images: ALOS-1 PALSAR images, Sentinel-1 SAR images, and high resolution images.

(1) Validation by ALOS-1 PALSAR images covering the entire SCS (2007–2010). Overlaying all detections on the detections from annual ALOS-1 PALSAR mosaic images (Supplementary Fig. S10c), we found 671 platforms were confirmed by four-phase PALSAR images (2007–2010), 704 by three-phase PALSAR images (2008–2010), 768 by two-phase PALSAR images (2009–2010), 828 by the 2010 PALSAR images, and the remaining 231 platforms were not confirmed, meaning that they may have been incorrectly detected, or were constructed between 2010 and 2015 after the acquisition date of PALSAR images. In addition, the time-series PALSAR images confirmed that 28 platforms were omitted.

(2) *Validation by up-to-date Setinel-1 SAR images along the coastal zones*. Overlaying all detections on the detections from 86 quasi-synchronous Sentinel-1 SAR images (Supplementary Fig. S10e) acquired in 2014–2015, we found 481 platforms were covered by Sentinel SAR images, and 19 were unsuccessfully detected (17 were also confirmed to be omitted by the ALOS validation) and 14 were incorrectly detected.

(3) Validation by high resolution images covering offshore oil/gas production agglomeration regions (2013–2015). A total of 46 2-m-resolution GF-images and 43 6-m-resolution ZY-3 images (Supplementary Fig. S10f), covering an area more than 1.55×10^5 km^2, were used. Among 768 platforms located in the regions covered by these high resolution images, 654 platforms were confirmed, remaining 114 platforms could not be confirmed: 98 cases were because of heavy cloud (among these, 78 cases were confirmed by ALOS-1 PALSAR images acquired in 2010) and 16 cases were because the platforms construction date lagged the high resolution image photograph date. In addition, a total of 23 platforms were not correctly identified (11 were also confirmed to be omitted by the ALOS/Setinel-1 validation), and 7 cases of noise were inadvertently retained (2 were also confirmed to be commissioned by the Setinel-1 validation).

Based on the above validation, a total of 42 offshore platforms were confirmed to be omitted and 19 were incorrectly retained, resulting in an omission error of 3.88 % and commission rate of 1.76 %. After correction, a total of 1082 offshore platforms in the SCS by March 2015 were enumerated. Due to the limit of space, here we only demonstrate the validation results in the Gulf of Thailand (Supplementary Fig. S18-S35, 17 regions) and the sea area claimed by Brunei (Supplementary Fig. S36-S43).

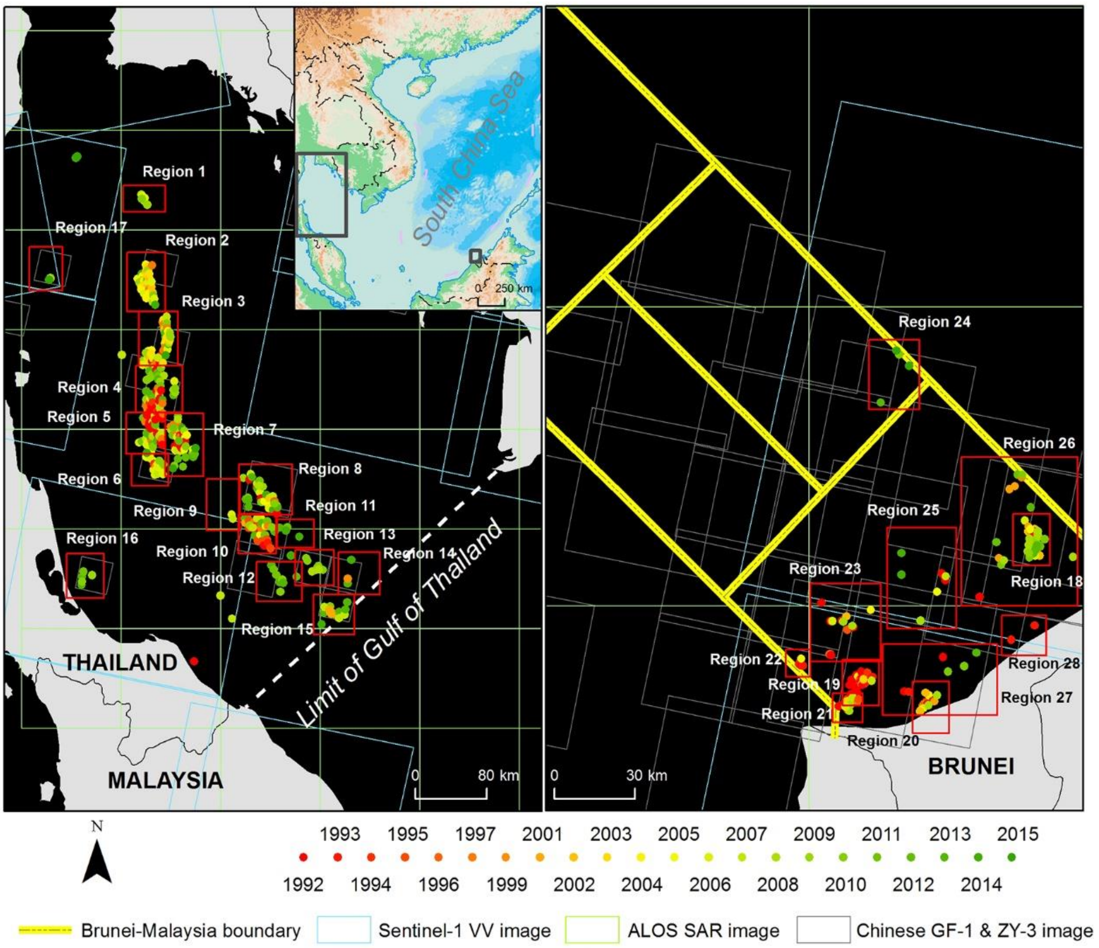

Supplementary Figure 18 | **Validation.** We crosschecked with all the platforms detected using other remote sensing data from multiple sources ranging from SAR to high-resolution optical images. Every offshore platform we detected has been convincingly evidenced by time-series satellite images. Due to the limit of space, here we only demonstrate the validation results in the sea area claimed by Brunei (right). More details about the validation across the Gulf of Thailand (left) please refer to the literature [30]. The figure was generated by Y.L. and J.S. using ArcMap 10.0 (http://www.esrichina.com.cn/).

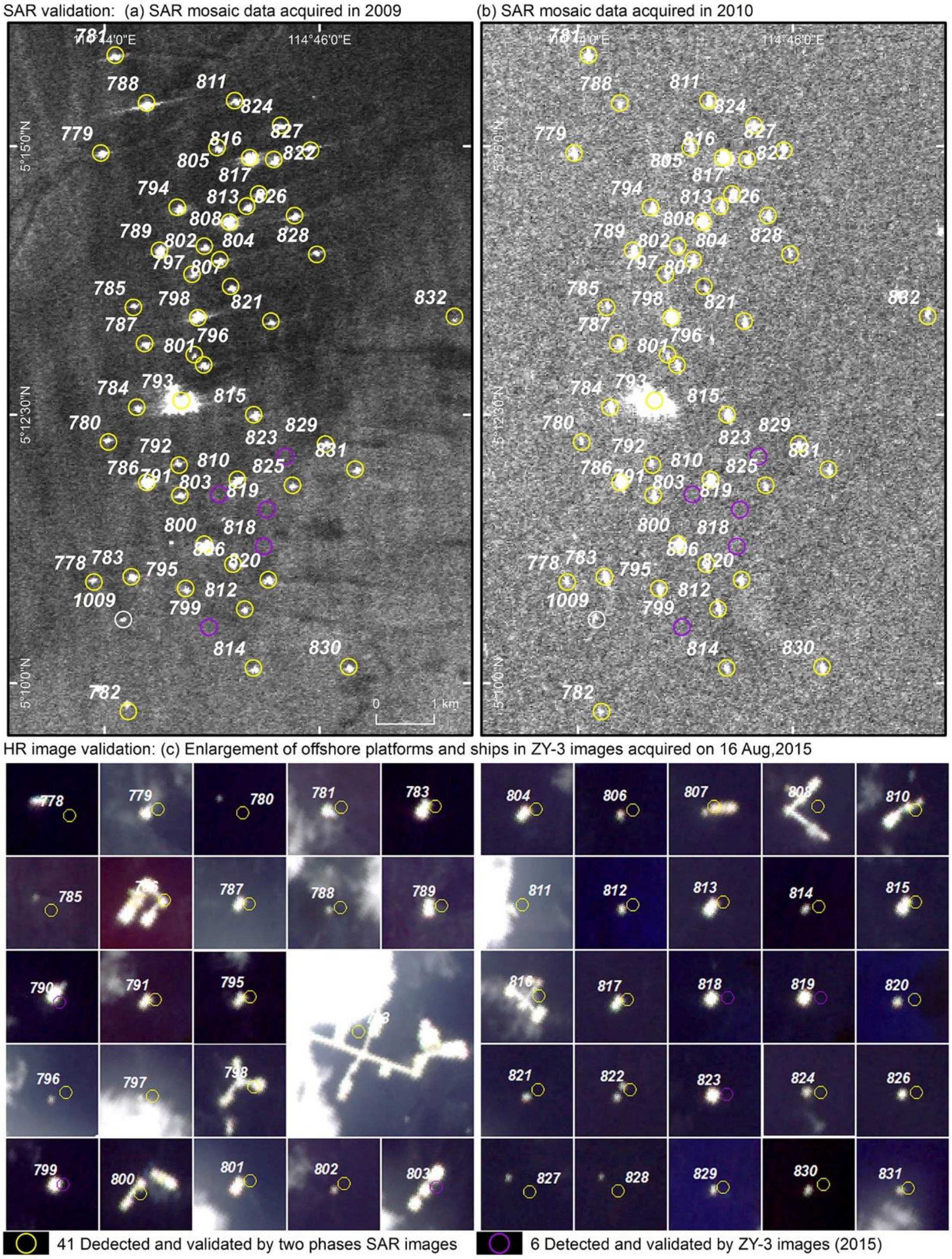

Supplementary Figure 19 | **Validation of the extracted offshore platforms in the sea area claim by Brunei** (Region 18). The GF-1 images were downloaded from the China Center for Resource Satellite Data and Applications (CRESDA, China, http://www.cresda.com/). The ALOS PALSAR Standard Products ((c) JAXA/METI) were downloaded from the Japan Aerospace Exploration Agency (JAXA, http://www.eorc.jaxa.jp/). The figure was generated by Y.L. and J.S. using ArcMap 10.0 (http://www.esrichina.com.cn/).

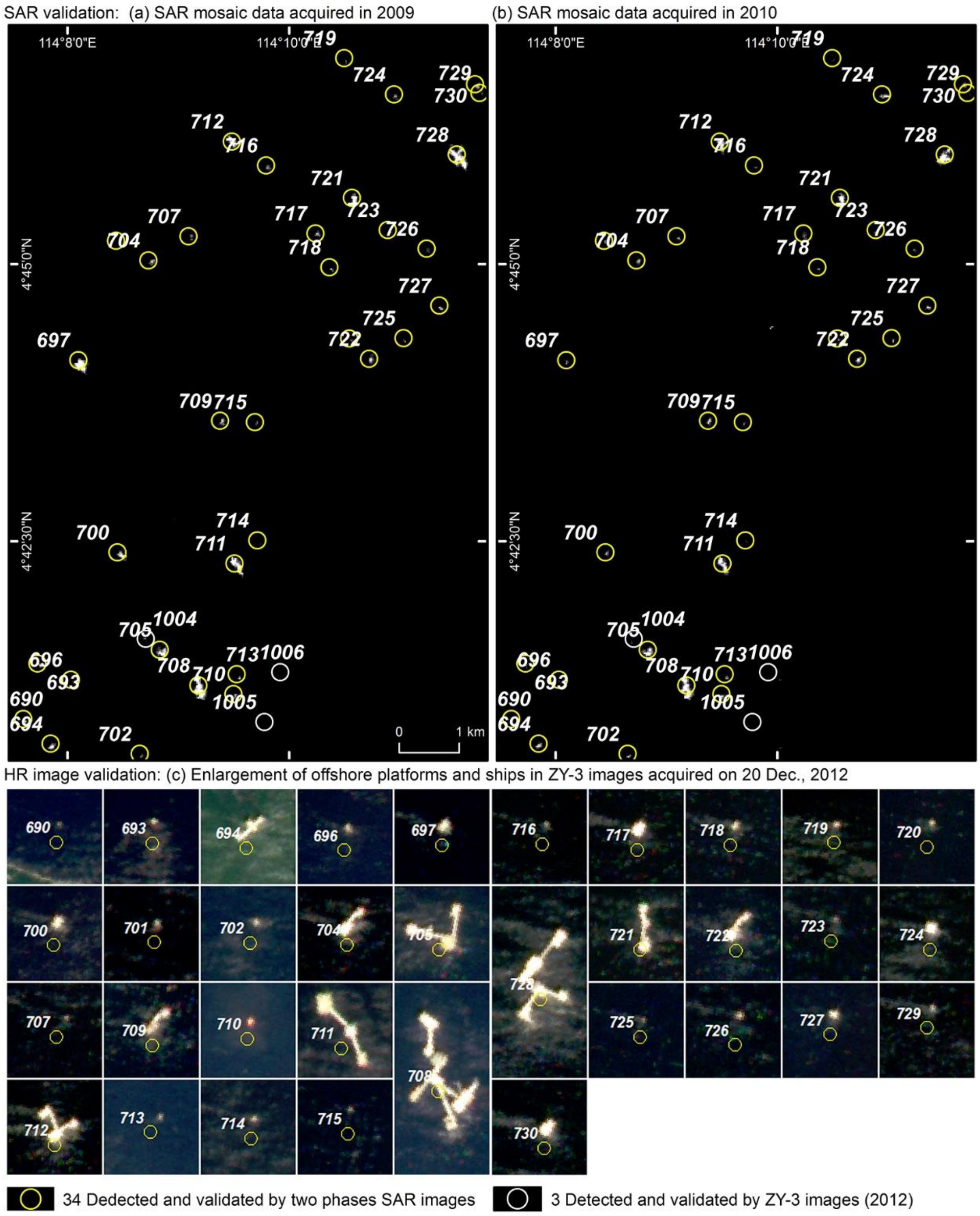

Supplementary Figure 20 | **Validation of the extracted offshore platforms in the sea area claim by Brunei** (Region 19). The GF-1 images were downloaded from the China Center for Resource Satellite Data and Applications (CRESDA, China, http://www.cresda.com/). The ALOS PALSAR Standard Products ((c) JAXA/METI) were downloaded from the Japan Aerospace Exploration Agency (JAXA, http://www.eorc.jaxa.jp/). The figure was generated by Y.L. and J.S. using ArcMap 10.0 (http://www.esrichina.com.cn/).

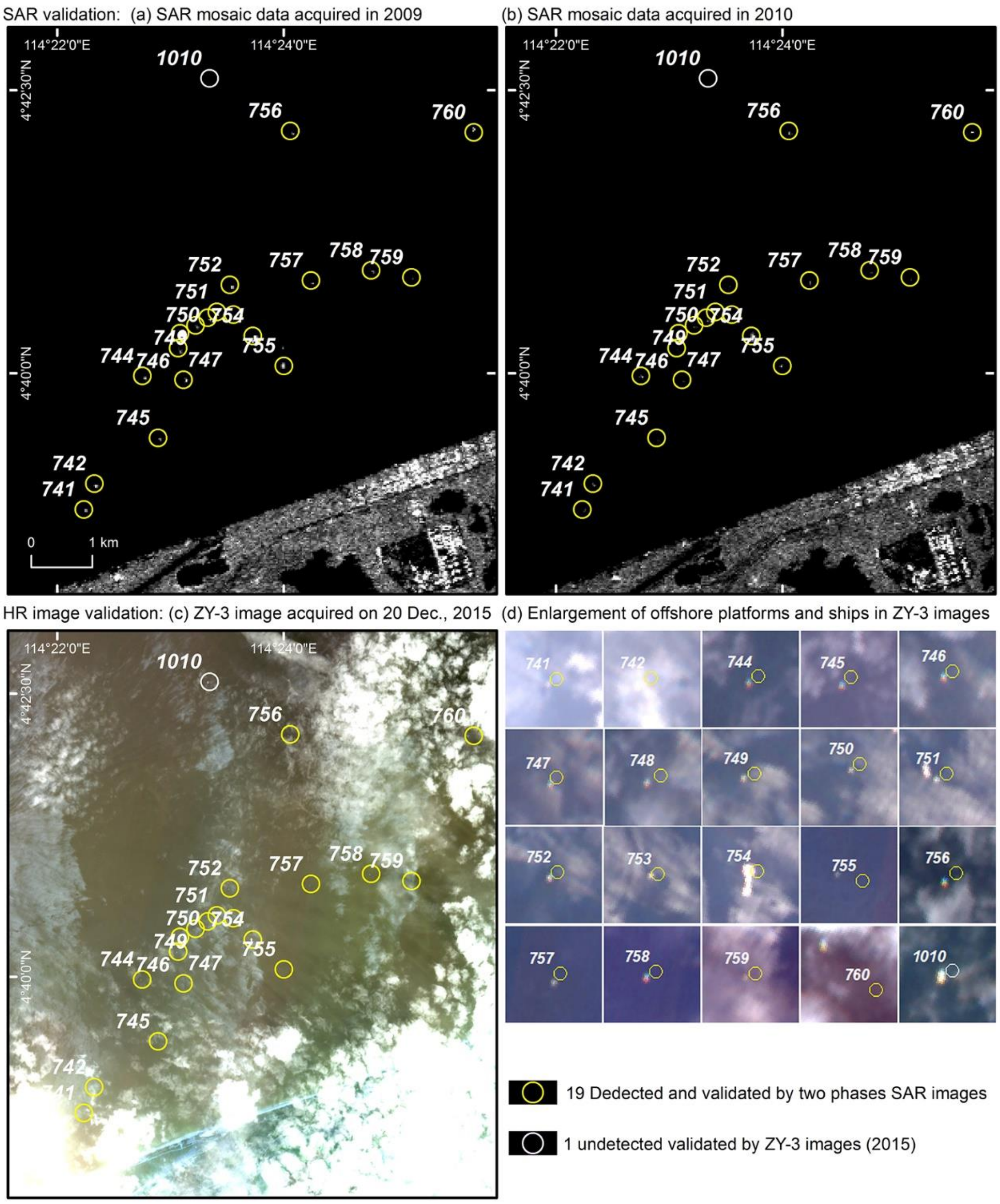

Supplementary Figure 21 | **Validation of the extracted offshore platforms in the sea area claim by Brunei** (Region 20). The GF-1 images were downloaded from the China Center for Resource Satellite Data and Applications (CRESDA, China, http://www.cresda.com/). The ALOS PALSAR Standard Products ((c) JAXA/METI) were downloaded from the Japan Aerospace Exploration Agency (JAXA, http://www.eorc.jaxa.jp/). The figure was generated by Y.L. and J.S. using ArcMap 10.0 (http://www.esrichina.com.cn/).

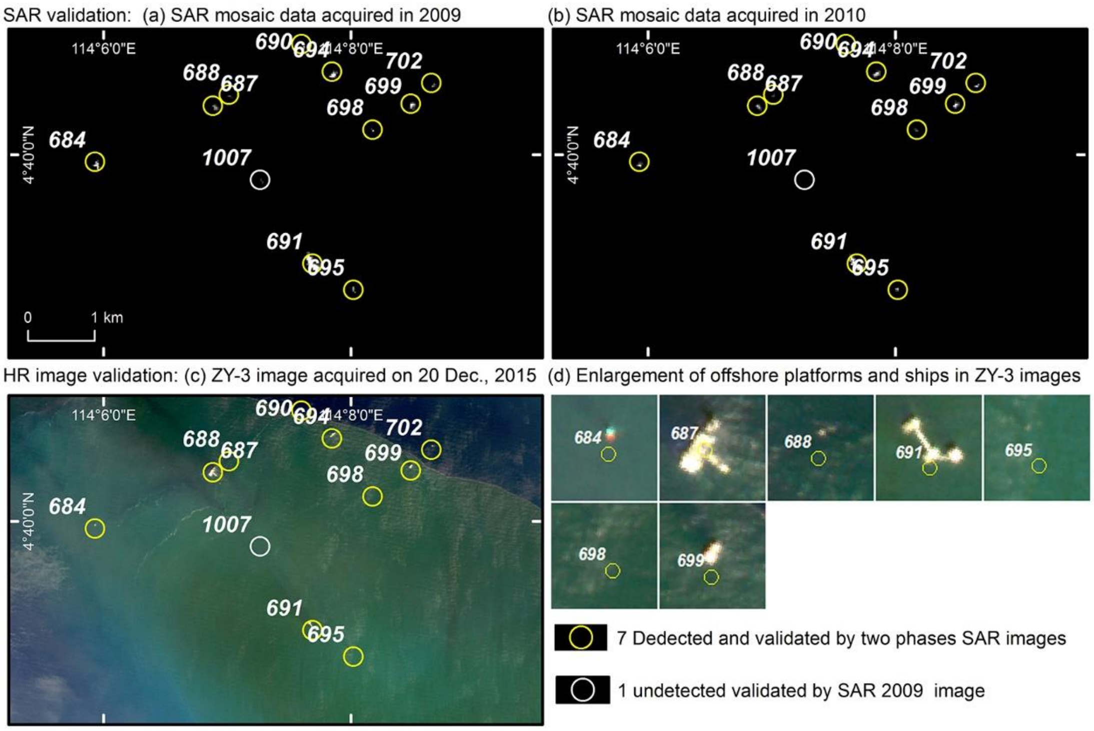

Supplementary Figure 22 | **Validation of the extracted offshore platforms in the sea area claim by Brunei** (Region 21). The GF-1 images were downloaded from the China Center for Resource Satellite Data and Applications (CRESDA, China, http://www.cresda.com/). The ALOS PALSAR Standard Products ((c) JAXA/METI) were downloaded from the Japan Aerospace Exploration Agency (JAXA, http://www.eorc.jaxa.jp/). The figure was generated by Y.L. and J.S. using ArcMap 10.0 (http://www.esrichina.com.cn/).

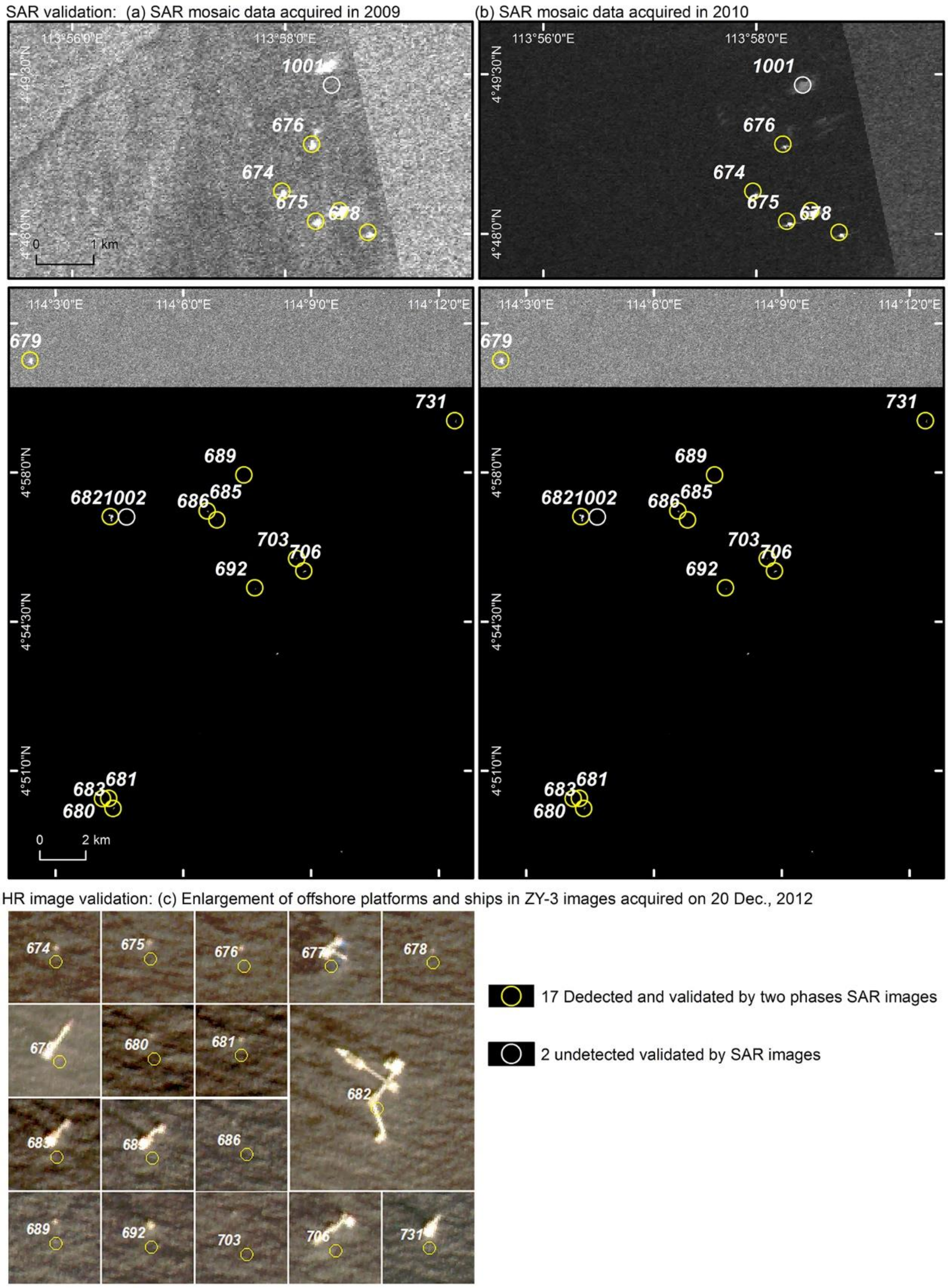

Supplementary Figure 23 | **Validation of the extracted offshore platforms in the sea area claim by Brunei** (Region 22). The GF-1 images were downloaded from the China Center for Resource Satellite Data and Applications (CRESDA, China, http://www.cresda.com/). The ALOS PALSAR Standard Products ((c) JAXA/METI) were downloaded from the Japan Aerospace Exploration Agency (JAXA, http://www.eorc.jaxa.jp/). The figure was generated by Y.L. and J.S. using ArcMap 10.0 (http://www.esrichina.com.cn/).

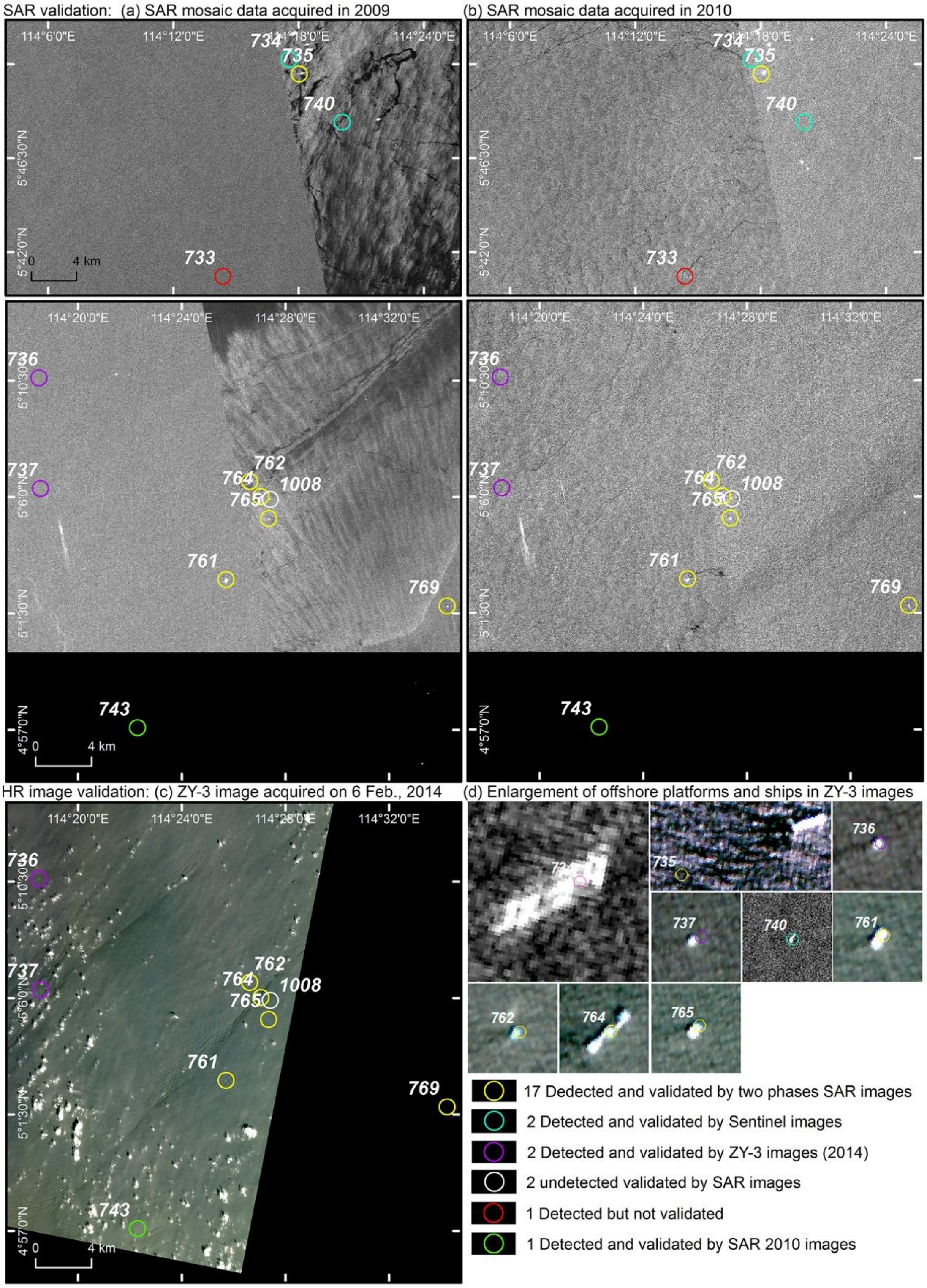

Supplementary Figure 24 | **Validation of the extracted offshore platforms in the sea area claim by Brunei** (Region 23). The GF-1 images were downloaded from the China Center for Resource Satellite Data and Applications (CRESDA, China, http://www.cresda.com/). The ALOS PALSAR Standard Products ((c) JAXA/METI) were downloaded from the Japan Aerospace Exploration Agency (JAXA, http://www.eorc.jaxa.jp/). The figure was generated by Y.L. and J.S. using ArcMap 10.0 (http://www.esrichina.com.cn/).

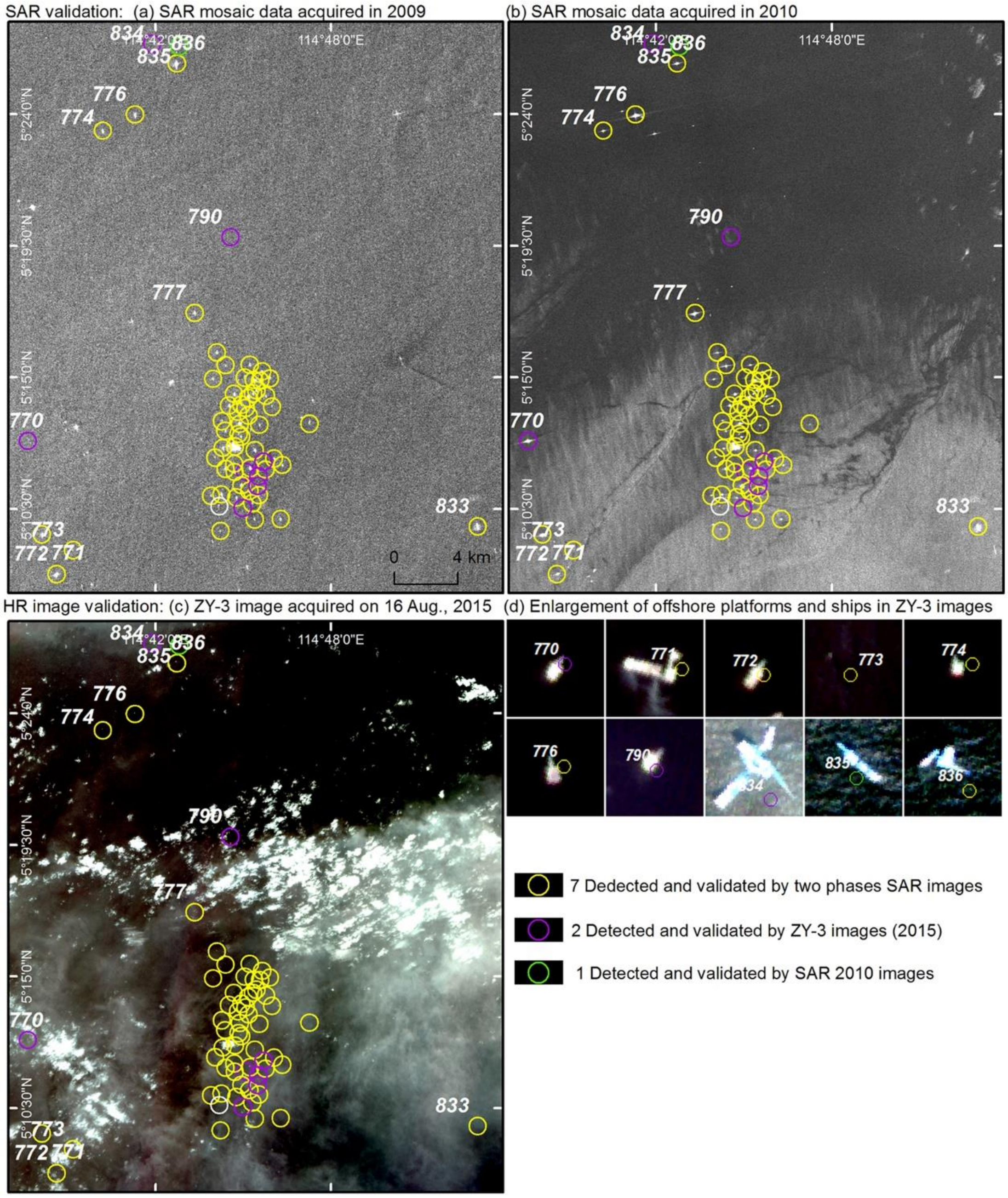

Supplementary Figure 25 | **Validation of the extracted offshore platforms in the sea area claim by Brunei** (Region 24). The GF-1 images were downloaded from the China Center for Resource Satellite Data and Applications (CRESDA, China, http://www.cresda.com/). The ALOS PALSAR Standard Products ((c) JAXA/METI) were downloaded from the Japan Aerospace Exploration Agency (JAXA, http://www.eorc.jaxa.jp/). The figure was generated by Y.L. and J.S. using ArcMap 10.0 (http://www.esrichina.com.cn/).

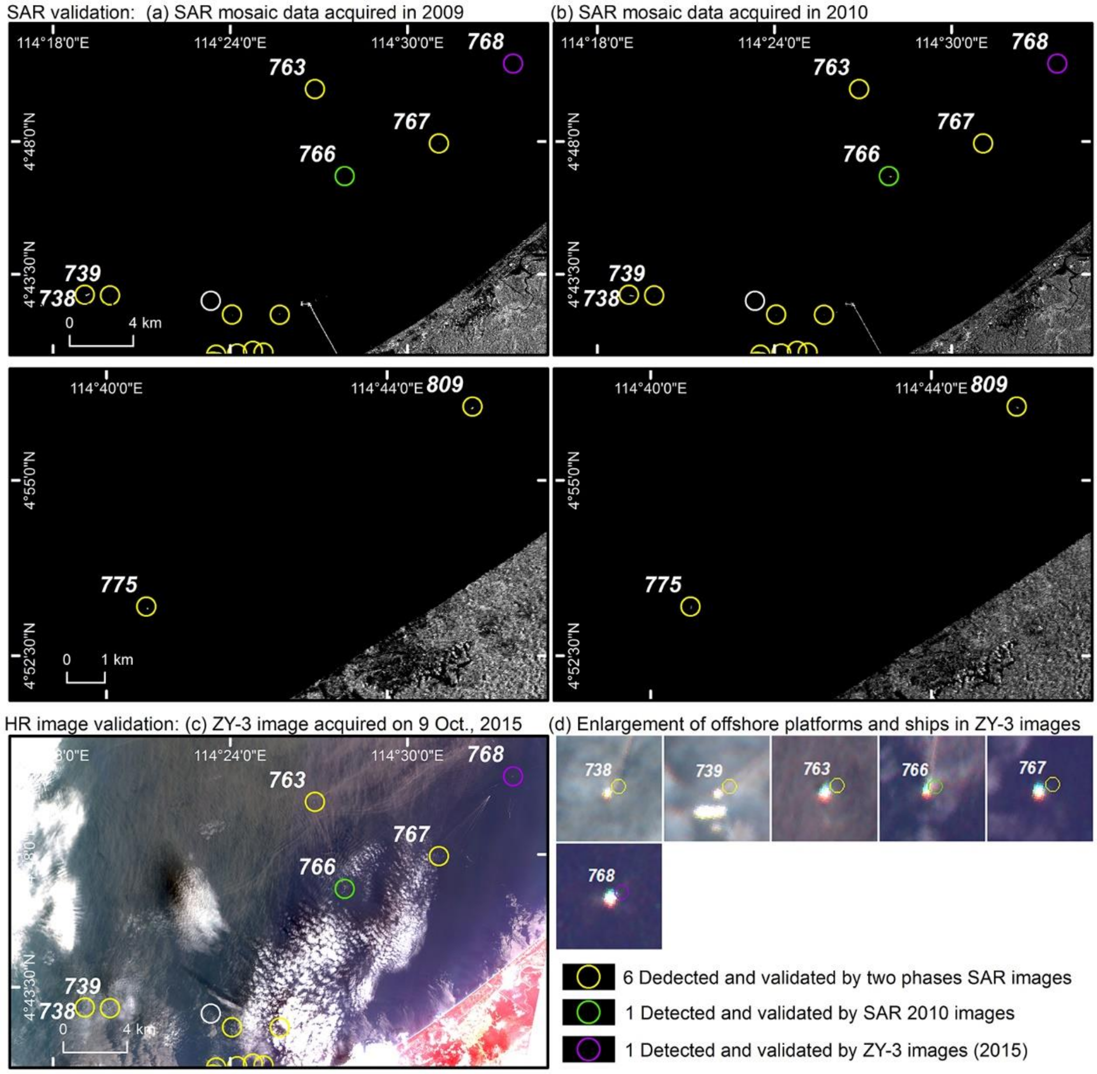

Supplementary Figure 26 | **Validation of the extracted offshore platforms in the sea area claim by Brunei** (Region 25，26, and 27). The GF-1 images were downloaded from the China Center for Resource Satellite Data and Applications (CRESDA, China, http://www.cresda.com/). The ALOS PALSAR Standard Products ((c) JAXA/METI) were downloaded from the Japan Aerospace Exploration Agency (JAXA, http://www.eorc.jaxa.jp/). The figure was generated by Y.L. and J.S. using ArcMap 10.0 (http://www.esrichina.com.cn/).

References

1. Morton, B. & Blackmore, G. South China Sea. *Mar Pollut Bull* **42,** 1236–1263 (2001).
2. Owen, N. A. & Schofield, C. H. Disputed South China Sea hydrocarbons in perspective. *Mar Policy* **36,** 809–822 (2012).
3. Lyons, Y. Prospects for satellite imagery of insular features and surrounding marine habitats in the South China Sea. *Mar Policy* **45,** 146–155 (2014).
4. Trung, N. N. The gas hydrate potential in the South China Sea. *J Petrol Sci Eng* **88,** 41–47 (2012).
5. Energy Information Administration. *South China Sea.* (2013) Available at: http://www.eia.gov/beta/international/regions-topics.cfm?RegionTopicID=SCS. (Accessed: 15th September 2014)
6. Dubois, E. P. Review of principal hydrocarbon-bearing basins of the South China Sea area. *Energy* **6,** 1113–1140 (1981).
7. Jin-Min, W. A history of oil and gas exploration in the central and northern parts of the South China Sea. *Energy* **10,** 413–419 (1985).
8. Saiful-Islam, A. B. M. *et al*. Review of offshore energy in Malaysia and floating Spar platform for sustainable exploration. *Renew Sust Energ Rev* **16,** 6268–6284 (2012).
9. Croft, T. A. Burning waste gas in oil fields. *Nature* **245,** 375–376 (1973).
10. Elvidge, C. D. *et al.* A fifteen year record of global natural gas flaring derived from satellite data. *Energies* **2,** 595–622 (2009).
11. Casadio, S., Arino, O. & Minchella, A. Use of ATSR and SAR measurements for the monitoring and characterisation of night-time gas flaring from off-shore platforms: The North Sea test case. *Remote Sens Environ* **123,** 175–186 (2012).
12. Casadio, S., Arino, O. & Serpe, D. Gas flaring monitoring from space using the ATSR instrument series. *Remote Sens Environ* **116,** 239–249 (2012).
13. Anejionu, O. C. D., Blackburn, G. A. & Whyatt, J. D. Detecting gas flares and estimating flaring volumes at individual flow stations using MODIS data. *Remote Sens Environ* **158,** 81–94 (2015).
14. Miller, S. D. *et al*. Suomi satellite brings to light a unique frontier of nighttime environmental sensing capabilities. *Proc Natl Acad Sci* 109, 15706–15711 (2012).
15. Haiyan, L., *et al*. Target detection on the ocean with the relative phase of compact polarimetry SAR. *IEEE T Geosci Remote* **51,** 3299–3305 (2013).
16. Arii, M., Koiwa, M. & Aoki, Y. Applicability of SAR to marine debris surveillance after the Great East Japan Earthquake. *IEEE J-STARS* **7,** 1729–1744 (2014).
17. Brekke C, Anfinsen SN. Ship detection in ice-infested waters based on dual-polarization SAR imagery. *IEEE Geosci Remote S* **8,** 391–395 (2011).
18. NOAA. *Version 4 DMSP-OLS Nighttime Lights Time Series.* (2012) Available from: http://ngdc.noaa.gov/eog/dmsp/downloadV4composites.html. (Accessed: 18th March 2015)
19. NOAA. *Version 1 Nighttime VIIRS Day/Night Band Composites.* (2014) Available from: http://ngdc.noaa.gov/eog/viirs/download_monthly.html. (Accessed: 18th March 2015)
20. NASA. *Landsat Science of OLI Landsat Science of OLI.* (2012) Available at: http://landsat.gsfc.nasa.gov/?p=3186. (Accessed: 18th March 2015)
21. USGS. *Landsat 8 Quality Assessment Band Landsat 8 Quality Assessment Band.* (2013) Available at:

http://landsat.usgs.gov/L8QualityAssessmentBand.php. (Accessed: 18th March 2015)

22. Storey, J., Choate, M. & Kenton, L. Landsat 8 Operational Land Imager on-orbit geometric calibration and performance. *Remote Sens* **6,** 11127–11152 (2014).
23. Gill, T. *et al.* Geometric correction and accuracy assessment of Landsat-7 ETM+ and Landsat-5 TM imagery used for vegetation cover monitoring in Queensland, Australia from 1988 to 2007. *J Spat Sci* **55,** 273–287 (2010).
24. Sertel, E., Kaya, S. & Ormeci, C. *Analysis of geometric accuracy of Landsat 5 TM and IRS 1D data by means of DGPS and Map data,* 2006.
25. Borgeson, W.T., Batson, R.M. & Kieffer, H. H. Geometric accuracy of Landsat-4 and Landsat-5 Thematic Mapper images. *Photogramm Eng Rem S* **51,** 1893–1898 (1985).
26. Shimada, M. *et al.* New global forest/non-forest maps from ALOS PALSAR data (2007–2010). *Remote Sens Environ* **155,** 13–31 (2014).
27. Shimada, M., Isoguchi, O., Tadono, T. & Isono, K. PALSAR radiometric and geometric calibration. *IEEE T Geosci Remote* **47,** 3915–3932 (2009).
28. Chen, N., Li, J. & Zhang, X. Quantitative evaluation of observation capability of GF-1 wide field of view sensors for soil moisture inversion. *J Appl Remote Sens* **9,** 097097–097097 (2015).
29. Xinming, T., Junfeng, X., Xiao, W. & Wanshou, J. High-precision attitude post-processing and initial verification for the ZY-3 Satellite. *Remote Sens* **7,** 111–134 (2015).
30. Liu, Y. X. *et al.* Automatic extraction of offshore platforms using time-series Landsat-8 Operational Land Imager data, *Remote Sens Environ*, 175, 73–91 (2016).
